# Rapid Investigation of Functional Roles of Genes in Regulation of Leaf Senescence Using Arabidopsis Protoplasts

**DOI:** 10.3389/fpls.2022.818239

**Published:** 2022-03-17

**Authors:** Phan Phuong Thao Doan, Jin Hee Kim, Jeongsik Kim

**Affiliations:** ^1^Interdisciplinary Graduate Program in Advanced Convergence Technology & Science, Jeju National University, Jeju, South Korea; ^2^Subtropical Horticulture Research Institute, Jeju National University, Jeju, South Korea; ^3^Faculty of Science Education, Jeju National University, Jeju, South Korea

**Keywords:** leaf senescence, protoplasts, transient expression, luciferase, cell death, ROS, *Arabidopsis thaliana*

## Abstract

Leaf senescence is the final stage of leaf development preceding death, which involves a significant cellular metabolic transition from anabolism to catabolism. Several processes during leaf senescence require coordinated regulation by senescence regulatory genes. In this study, we developed a rapid and systematic cellular approach to dissect the functional roles of genes in senescence regulation through their transient expression in Arabidopsis protoplasts. We established and validated this system by monitoring the differential expression of a luciferase-based reporter that was driven by promoters of *SEN4* and *SAG12*, early and late senescence-responsive genes, depending on effectors of known positive and negative senescence regulators. Overexpression of positive senescence regulators, including *ORE1*, *RPK1*, and *RAV1*, increased the expression of both *SEN4*- and *SAG12-LUC* while *ORE7*, a negative senescence regulator decreased their expression. Consistently with overexpression, knockdown of target genes using amiRNAs resulted in opposite *SAG12-LUC* expression patterns. The timing and patterns of reporter responses induced by senescence regulators provided molecular evidence for their distinct kinetic involvement in leaf senescence regulation. Remarkably, *ORE1* and *RPK1* are involved in cell death responses, with more prominent and earlier involvement of *ORE1* than *RPK1*. Consistent with the results in protoplasts, further time series of reactive oxygen species (ROS) and cell death assays using different tobacco transient systems reveal that *ORE1* causes acute cell death and *RPK1* mediates superoxide-dependent intermediate cell death signaling during leaf senescence. Overall, our results indicated that the luciferase-based reporter system in protoplasts is a reliable experimental system that can be effectively used to examine the regulatory roles of Arabidopsis senescence-associated genes.

## Introduction

Leaf senescence is the final and degenerative stage of a leaf’s life history; however, it is necessary for plant succession as a beneficial developmental process. Plants coordinate energy and nutrient use among individual leaves when they remobilize resources to newly developing organs or offspring by inducing leaf senescence in old leaves or those having a low photosynthetic efficiency. Plants determine the fate of leaves to be senesced by reducing photosynthetic activity, dismantling chloroplasts, and changing color, often from green to yellow and/or red. During leaf senescence, the redistribution of nutrients and energy is initially derived from the gradual breakdown of chloroplasts, followed by the catabolism of macromolecules, such as nucleic acids, proteins, and lipids, and degeneration of mitochondria and nuclei (Lim et al., 2007a). For their efficient remobilization, various types of transporters for the molecules composed of carbon, nitrogen, and phosphate are activated (Stigter and Plaxton, 2015; Have et al., 2017). Furthermore, plant leaves sustain self-maintenance activities, such as pathogen defense and detoxification of reactive oxygen species to complete their redistribution activities (Lim et al., 2003; Koyama, 2018).

Plants evolve sophisticated genetic programs for determining the appropriate senescence onset and coordinating senescence progression. The onset of senescence is triggered by various endogenous and environmental signals through the coordinated actions of multiple senescence induction pathways (Woo et al., 2019; Camargo Rodriguez, 2021). A well-studied genetic pathway for senescence onset in Arabidopsis is the trifurcate death circuit consisting of ORE1, EIN2 as an ORE1 activator, and miRNA164 as an ORE1 repressor. ORE1 is a crucial genetic factor to determine leaf senescence onset, and its activation in an aged tissue is inevitable due to EIN2-mediated direct activation or release of miRNA164 repression (Kim et al., 2009). Another example is the protein trio RPK1-CaM4-RbohF, which regulates the transient superoxide production to trigger age- and ABA-dependent leaf senescence and cell death (Lee et al., 2011; Koo et al., 2017).

The onset of leaf senescence by senescence regulatory genes induces changes in the expression of diverse executive senescence-associated genes (SAGs) for the systemic progression of biochemical and physiological processes during leaf senescence. For example, *SEN4* and *SAG12* encode xyloglucan endotransglucosylase/hydrolase 24 and cysteine protease, respectively, which are mainly involved in macromolecule degradation and are upregulated during senescence (Woo et al., 2001; Lim et al., 2003). Conversely, the expression of chlorophyll a/b binding protein gene and rubisco small subunit gene encoding the subunit of light-harvesting complex and rubisco, respectively, declines with senescence progression (Woo et al., 2001). The senescence-associated expression of SAGs is coordinated by a time-dependent involvement of multiple positive and negative senescence regulatory elements, including transcription factors, ncRNA, and signaling components (Woo et al., 2016). Members of NAC and WRKY, two of major senescence-associated transcription factor families are expressed with various temporal patterns along with aging and play positive or negative roles in senescence regulation by regulating the timely expression of SAGs, including genes in the same gene family (Hickman et al., 2013; Kim et al., 2014; Woo et al., 2016; Li et al., 2018). Additionally, a comprehensive study investigating the temporal involvement of senescence regulators identified dynamic changes in NAC regulatory hubs along with leaf aging (Kim et al., 2018a). Time-course expression of NACs in 49 NAC mutants revealed the transition of NAC hubs and their regulatory modules from mature to middle senescent stages and NAC troika was highlighted as a critical hub at the presenescent stage that predominantly repressed the expression of SAGs involved in SA- and reactive oxygen species (ROS) dependent responses.

The temporal and kinetic response analyses aid in understanding gene properties and functions, which further help to elucidate the functional role of genetic pathways (Kim et al., 2018b; Woo et al., 2019). Transient gene expression assays using leaf mesophyll protoplasts are widely used as one of the most efficient approaches for characterizing the cellular functions and regulatory networks of genes in plants in a relatively short time (Rolland, 2018; Domozych et al., 2020). It has contributed to the dissection of signaling pathways in responses to plant hormones or environmental factors (Hwang and Sheen, 2001; Yoo et al., 2007; Li et al., 2019; Lehmann et al., 2020). Recently, knockdown approaches using either RNAi or artificial microRNA (amiRNA) have enabled a reduction in the expression of the endogenous target gene in protoplasts, extending the application of this technology to evaluate gene knockdown effects (Ossowski et al., 2008; Kim and Somers, 2010; Zhang et al., 2019; Vachova et al., 2020). Furthermore, the protoplast viability extension has enabled the investigation of long-term kinetic molecular responses using a luciferase-based reporter for circadian biology (Kim and Somers, 2010). Although leaf senescence is recognized as a long-term developmental event that is controlled by a complex temporal interaction of regulatory components, kinetic analyses using protoplasts have not been applied to plant senescence studies.

In this study, we established a rapid and efficient approach to rapidly dissect the functional roles of genes in leaf senescence regulation through their transient expression using Arabidopsis mesophyll protoplasts. We used overexpression and knockdown approaches to guide the expression of target genes and discovered an altered expression of target genes and SAG reporters, demonstrating its feasibility for investigating a potential regulatory role of senescence regulatory genes at the cellular level. Moreover, these approaches, coupled with histochemical analysis, can reveal distinct and convergent *ORE1* and *RPK1* functions in mediating ROS responses during leaf senescence.

## Materials and Methods

### Plant Materials and Growth Conditions

Arabidopsis (*Arabidopsis thaliana*) Col-0 wild-type and tobacco (*Nicotiana benthamiana*) plants were sown in pots and grown in an environmentally controlled culture room under LD conditions at 22°C (16 h light/8 h dark cycle; cool white fluorescent bulb with 100–150 μmol m^2^ s^−1^; TLD/840RS; and Philips).

### Plasmid Construction

We generated plasmid constructs for transient gene expression in protoplasts or tobacco using GATEWAY cloning technology (Invitrogen, United States). For the reporter plasmids (*SEN4*- and *SAG12-LUC*), the 5′ upstream regions encompassing the *SEN4* (At4g30270) and *SAG12* (At5g45890) promoters of 1.38 kb and 1.53 kb in length, respectively, were amplified by polymerase chain reaction (PCR) with Pfu DNA polymerase, using Arabidopsis Col-0 genomic DNA as a template and appropriate sets of primers (Supplementary Table 1). We subcloned the amplified DNA fragments into the entry vector of pCR-CCD-R using the corresponding restriction enzymes to produce entry clones. The promoter-LUC final constructs were established by LR recombination using the corresponding entry clones and gateway version of the pOmegaLUC_SK+ vector (Kim and Somers, 2010). We used a renilla luciferase (RLUC) under the control of 35S promoter (35S-RLUC) as the transfection control. For the overexpression effectors, the full-length coding sequence of *ORE1* (At5g39610), *ORE7* (At1g20900), *RAV1* (At1g13260), and *RPK1* (At1g69270) was amplified using PCR from Arabidopsis cDNA pools with Pfu DNA polymerase and gene-specific primers (Supplementary Table 1). Further, we subcloned the amplified DNA fragments into the pCR-CCD-F entry vector to produce entry clones. Then, we recombined the entry clones using gateway versions of pCsVMV-eGFP-N-999 and pCsVMV-eGFP-N-1300 to produce the effector plasmids of ORE1-, RAV1-, RPK1-, and ORE7-pCsVMV-eGFP-N-999 and binary plasmids of ORE1-, RAV1-, RPK1-, and ORE7-pCsVMV-eGFP-N-1300, respectively. For amiRNA effector, ORE1 amiRNA plasmid was generated by digesting pAmiR-ORE1 (CSHL_075023) with *Pst*I and *Bam*HI, then, ligating each resulting amiRNA foldback fragment into *Pst*I/*Bam*HI digested pCsVMV-PP2C-AmiR vector (Kim and Somers, 2010). We designed RPK1 amiRNA using the Web MicroRNA Designer 3^1^ as previously described (Schwab et al., 2006; Kim and Somers, 2010). The amiRNA foldback fragments were generated by overlapping PCR using the pCsVMV-PP2C-AmiR plasmid as a template and the designated primers for each construct (Supplementary Table 1). All resulting PCR fragments containing the full amiRNA foldback were cloned downstream of the CsVMV promoter into unique *Pst*I and *BamH*I restriction sites of pCsVMV-AmiR. Also, we utilized the pCsVMV-AmiR plasmid as a transfection control.

### Protoplast Isolation and Transfection

We conducted protoplast isolation and DNA transfection as previously described (Kim and Somers, 2010). Briefly, 10 to 15 leaves of three- to four-week-old Col-0 plants were sterilized with 70% ethanol for 30 s, and then rinsed with sterile water twice. Leaves scratched briefly with sandpaper were incubated in 10 ml of enzyme solution (1% Cellulase R10, 0.5% Macerozyme R10 [Yakult Honsha, Japan], 400 mM mannitol, 20 mM KCl, 10 mM CaCl_2_, 20 mM MES-KOH [pH 5.7], and 0.1% BSA [Sigma A6793, United States]) for 2.5 h by gentle shaking at room temperature. Protoplasts released into enzyme solutions were filtered and harvested into a round-shaped culture tube by centrifugation at 100 g for 5 min. We resuspended the protoplast pellets in 2 ml of W5 solution (154 mM NaCl, 125 mM CaCl_2_, 5 mM KCl, 1.5 mM MES-KOH [pH 5.7], and 5 mM Glucose), and placed them on ice for 30 min. The protoplasts were harvested and resuspended in MMG solution (400 mM mannitol, 15 mM MgCl_2_, and 4 mM MES-KOH [pH 5.7]), and the final cell concentration was adjusted to 2 × 10^5^ ml^−1^. Also, 30.2 μl of plasmid mixtures with 25 μl effector, 5 μl reporter, and 0.2 μl internal control were transfected into 200 μl of protoplasts in MMG solution. We prepared effector plasmids of overexpression or amiRNA and reporter plasmids by CsCl gradient purification using an ultracentrifuge (in Bio-Health Materials Core-Facility, Jeju National University, Korea), and their DNA concentration was adjusted to 2 μg μl^−1^ per 4 kb DNA. We performed transfections by adding 230 μl (1 vol.) of polyethylene glycol (PEG) solution [40% PEG-4000, 200 mM mannitol, and 100 mM Ca(NO_3_)_2_] into protoplasts containing DNAs, and incubating them for 8–15 min at room temperature. We diluted the protoplast-DNA-PEG mixture with 920 μl (2 vol.) of W5 solution. After centrifugation, the pellets were resuspended in 700 μl of W5 solution containing 5% fetal bovine serum (Sigma F4135, United States) and 50 μg ml^−1^ ampicillin. For RT-PCR analysis from protoplasts, we used 800 μl of protoplasts and 120 μl of amiRNA plasmids for a transfection sample.

### Luminescence Measurement

We analyzed the expression of the specific senescence reporters by kinetic measurement of luciferase activity in protoplasts. A 300 μl of transfected protoplasts were transferred into each well containing 3 μl of LUC substrate (5 mM luciferin, Goldbio LUCK-250, Netherlands) or 3 μl of RLUC substrate (10 μM Coelenterazine-native, Sigma C2230, United States) of a white and round bottom 96-well microplate. The microplate was covered with a clear plastic cap and incubated at 22°C in the dark on a GloMax 96 microplate luminometer (Promega, United States). For 3 days, we acquired images every 30 min. In each data set, we determined promoter activities by luciferase activity at the indicated time and normalized them to the maximum RLUC level throughout the measurement. The relative LUC expression was calculated by normalizing to the maximum level of LUC/RLUC throughout the measurement in protoplasts transfected with GFP or the control amiRNA effector during each trial.

### RNA Extraction and Quantitative Reverse Transcriptase-PCR

We conducted total RNA extraction and cDNA synthesis, as previously described (Kim and Somers, 2010). Protoplasts were incubated for 16 h after transfection under dim white light and flash-frozen in liquid nitrogen for subsequent RNA extraction and qRT-PCR. Then, we extracted total RNA using WelPrep™ Total RNA Isolation Reagent (Welgene, Korea) and used it for cDNA synthesis using the ImProm II™ Reverse Transcriptase System kit (Promega, United States). Further, qPCR was performed on a CFX96 real-time qPCR detection system (Bio-Rad, United States) using appropriate primer sets. We designed primers for candidate genes using Primer 3 software (Untergasser et al., 2012) and are listed (Supplementary Table 1). The relative expression of target genes was calculated using the 2^−ΔΔCT^ method (Kim et al., 2008). *ACT2* gene was used as the internal reference.

### Transient Expression in Tobacco and Histochemical Analysis

We conducted a transient expression in tobacco using P19-enhanced Agrobacteria infiltration (Voinnet et al., 2003). Agrobacteria containing plasmids of ORE1- or RPK1-pCsVMV-eGFP-N-1300 or empty pCsVMV-eGFP-N-1300 were infiltrated into tobacco. Tobacco leaves were incubated at 22°C in the same chamber where plants are cultivated before being harvested on the days indicated after infiltration. Next, we performed a histochemical analysis with minor modifications as described (Yu et al., 2019). For the visualization of H_2_O_2_ and superoxide accumulation, 3,3′-diaminobenzidine (DAB; Sigma D8001, United States) and nitrotetrazolium blue chloride (NBT; Sigma N6639, United States) were used, respectively. Tobacco leaves transiently expressing *ORE1-GFP*, *RPK1-GFP*, and *GFP* were subjected to DAB and NBT staining at the indicated days. The leaves were soaked in 1 mg ml^−1^ DAB or 2 mg ml^−1^ NBT overnight and boiled for 10 min in 100% ethanol. Stained leaves were stored in 95% ethanol at room temperature before being photographed. Cell death in tobacco leaves was visualized by trypan blue staining. The treated tobacco leaves were completely immersed in 1 mg ml^−1^ trypan blue (Fluka 93590, Switzerland) solution and incubated for at least 30 min at room temperature. The stained leaves were washed immediately with 98–100% ethanol for decolorization and photographed under a bright-field microscope. Intensities of trypan blue, DAB, or NBT staining were quantified using ImageJ software^2^ as previously described (Juszczak and Baier, 2014). Stained leaf disks from five leaves expressing *GFP*, *ORE1-GFP*, and *RPK1-GFP* were then used to quantify staining intensity.

### Protoplast Viability Test

Cell death of Arabidopsis protoplasts was assayed by Evans blue staining (Sigma E2129, United States). The protoplasts expressing *GFP*, *ORE1-GFP*, *RAV1-GFP*, *RPK1-GFP*, and *ORE7-GFP* were incubated for 72 h under dim white light. We extracted transfected protoplasts at different time points (0, 6, 24, 48, and 72 h after transfection) and loaded them for 2–3 min with 10 mg ml^−1^ Evans blue dye. We visualized and photographed blue-stained dead cells using Zeiss Axiostar Plus Microscope (Ambastha et al., 2017). Stained protoplasts were counted in five to twelve fields containing at least 50 cells from each sample cell death measurement. Cell death (%) was measured as the following formula: Number of blue cells/Total number of cells × 100%.

## Results

### Establishment of Luciferase-Based Reporters Controlled by the Promoter of SAGs for Cell-Based Senescence Assay

**Figure 1:**
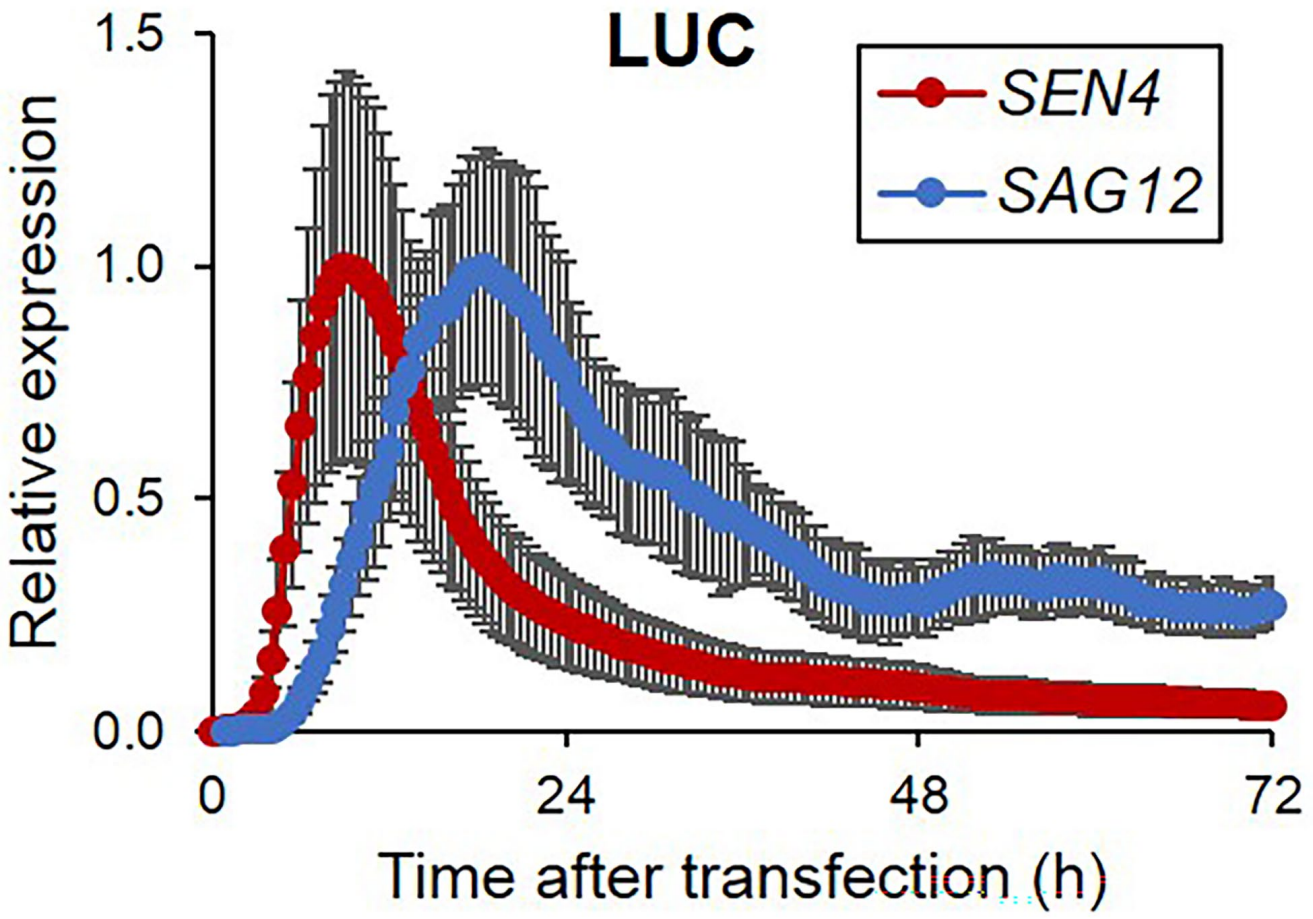
Bioluminescence expression patterns of luciferase reporters controlled by senescence-associated promoters in Arabidopsis protoplasts during darkness. Bioluminescence traces in Arabidopsis protoplasts expressing a firefly luciferase gene (*LUC*) driven by *SEN4* and *SAG12* promoters (*SEN4*- and *SAG12-LUC*) and a Renilla luciferase (RLUC) driven by 35S promoter (35S-RLUC) in darkness after transfection are shown. Image acquisition was performed every 30 min for 3 d. Luciferase activity of LUC was normalized to the maximum level of RLUC over the assay. Each data set was normalized to the maximum level of LUC/RLUC throughout the measurement. Data represent mean ± SE (*n* = 3). Similar results were obtained in two independent trials.

Arabidopsis protoplasts are an effective experimental system for rapid functional analyses, enabling us to investigate diverse molecular and cellular functions of genes of interest based on responsiveness luciferase reporters through their transient expression (Tyurin et al., 2020). Firstly, to establish the luciferase-based reporters for investigating leaf senescence in protoplasts, we selected two SAGs, including *SAG12* and *SEN4* with increased expression during the dark- and age-induced leaf senescence. Each SAG promoter-driven luciferase reporter construct was transfected individually into Arabidopsis mesophyll protoplasts and we monitored luminescence activity in a time-series manner. As shown in Figure 1, the expression of *SEN4-LUC* and *SAG12-LUC* was significantly induced with different accumulation rates and peak periods but was quickly reduced following their peak expression. *SEN4-LUC* expression was induced more rapidly with an earlier peak time than *SAG12-LUC*, which is similar to that observed in intact leaves (Supplementary Table 2; Woo et al., 2001, 2002). Additionally, expression levels of *SEN4*- and *SAG12*-*LUC* were higher than that obtained from the basal expression level of a promoterless-*LUC* reporter (Supplementary Figure 1). Peaks of their expression were also different from *35S*-*LUC*, thereby supporting the potential use of *SEN4*- and *SAG12*-*LUC* as cellular senescence reporters. We also tested other *LUC* reporters driven by the promoters of *PRK*-, *CA1*-, and *THIONIN-LUC* with downregulated expression during senescence but failed to obtain reliable expression patterns for a reporter assay, although their transcript levels are high in transcriptome analysis during age- and dark-induced senescence (Supplementary Figure 1; Woo et al., 2016; Kim et al., 2018c). From these results, we conducted subsequent analyses using only the *SEN4-LUC* and *SAG12-LUC* reporters.

### Transient Overexpression of Senescence Regulatory Genes as Effectors

**Figure 2:**
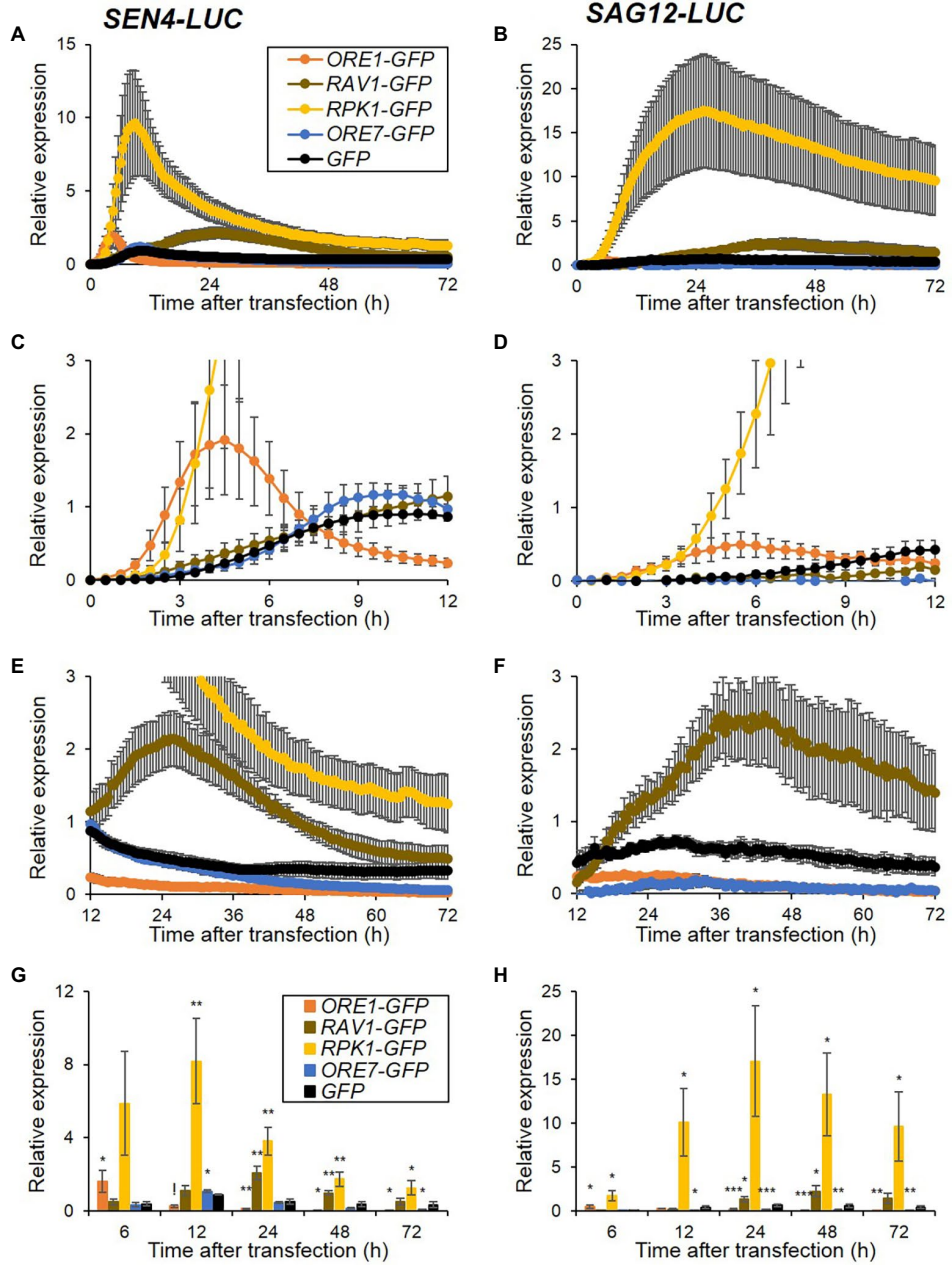
Expression patterns of *SEN4*- **(A)** and *SAG12-LUC* **(B)** reporters by overexpression of senescence regulators. Arabidopsis protoplasts were co-transfected, as described in Figure 1, but with overexpression effectors of *GFP*, *ORE1*-, *RAV1*-, *RPK1*-, and *ORE7-GFP* and either reporter of *SEN4*- or *SAG12-LUC*. **(A–F)** Bioluminescence traces of *SEN4*- or *SAG12-LUC* throughout the measurement **(A,B)**, and their traces with an adjusted scale at early (0–12 h; **C,D**) and late (12–72 h; **E,F**) time points are shown. Symbols in **(B–F)** are the same as in **(A)**. Luciferase activity of LUC was normalized to the maximum level of RLUC over the assay. Relative expression of *SEN4*- **(A,C,E)** and *SAG12-LUC* **(B,D,F)** was calculated by normalization to the maximum level of LUC/RLUC throughout the measurement in protoplasts transfected with *GFP* effector in each trial. **(G,H)** Relative expression of *SEN4*- **(G)** and *SAG12-LUC* **(H)** at 6, 12, 24, 48, and 72 h after transfection. Symbols in **(H)** are the same as in **(G)**. Data represent mean ± SE (*n* = 6). A statistical analysis was performed using a two-tailed Student’s *t*-test (^*^p < 0.05; ^**^p < 0.01; ^***^p < 0.001; and ^!^*p* < 0.0001).

Since the *SEN4*- and *SAG12-LUC* revealed clear and distinct expression patterns in protoplasts, we attempted to evaluate the effect of ectopic overexpression of senescence regulatory genes on the expression of both reporters. We generated overexpression effector constructs for positive (*ORE1*, *RAV1*, and *RPK1*) and negative (*ORE7*) senescence regulators fused to a green-fluorescent protein (GFP) under the control of Cassava vein mosaic virus (CsVMV) promoter. We confirmed that these effector proteins were strongly expressed and exclusively localized in subcellular organelles as reported; ORE1, RAV1, and ORE7 in the nucleus and RPK1 in the plasma membrane (Supplementary Figure 2; Lim et al., 2007b; Kim et al., 2009; Woo et al., 2010; Koo et al., 2017). GFP-fused senescence regulator effector plasmids and GFP as a control were co-transfected with *SEN4-LUC* and *SAG12-LUC* reporters, and time-series luminescence levels of the reporters were compared (Figure 2). When *GFP* was transiently introduced, the luminescence expression of both reporters was induced with differential expression levels and patterns throughout the assay for 72 h. Ectopic expression of *RPK1* led to a 10- and 18-fold increase in *SEN4-LUC* and *SAG12-LUC* expression at the peak time, respectively, but no significant change in their peak time compared with control *GFP* expression (Figures 2A,B; Supplementary Table 2). Interestingly, ectopic expression of *ORE1* induced distinct expression patterns of *SEN4*- and *SAG12-LUC* in that their expression was induced earlier and reached maximum levels more rapidly, compared with that of control, and was dampened quickly (Figures 2C,D,G,H; Supplementary Table 2). However, *RAV1* had a late inducing effect on the expression of *SEN4-LUC* and *SAG12-LUC*, and maintained their expression higher, although both expressions earlier were the same or lower relative to those of the control (Figures 2E–H; Supplementary Table 2). Overexpression of *ORE7*, as a negative senescence regulator, resulted in a dampened expression of *SAG12-LUC* throughout the assay, but a slight reduction of *SEN4-LUC* expression at a later time of assay ranging from 65 to 72 h (Figures 2E *–*H). These results were consistent with early senescence phenotypes in *ORE1*, *RPK1*, and *RAV1* overexpressors, and delayed senescence phenotypes in *ORE7* overexpressor (Lim et al., 2007b; Kim et al., 2009; Woo et al., 2010; Koo et al., 2017). Hence, our result indicates that the effectiveness of senescence regulators can be evaluated by overexpressing them, and co-expressing *SEN4-LUC* and *SAG12-LUC* reporters through a prolonged protoplast assay.

### Transient amiRNA-Mediated Knockdown of the Senescence Regulatory Genes as Effectors

**Figure 3:**
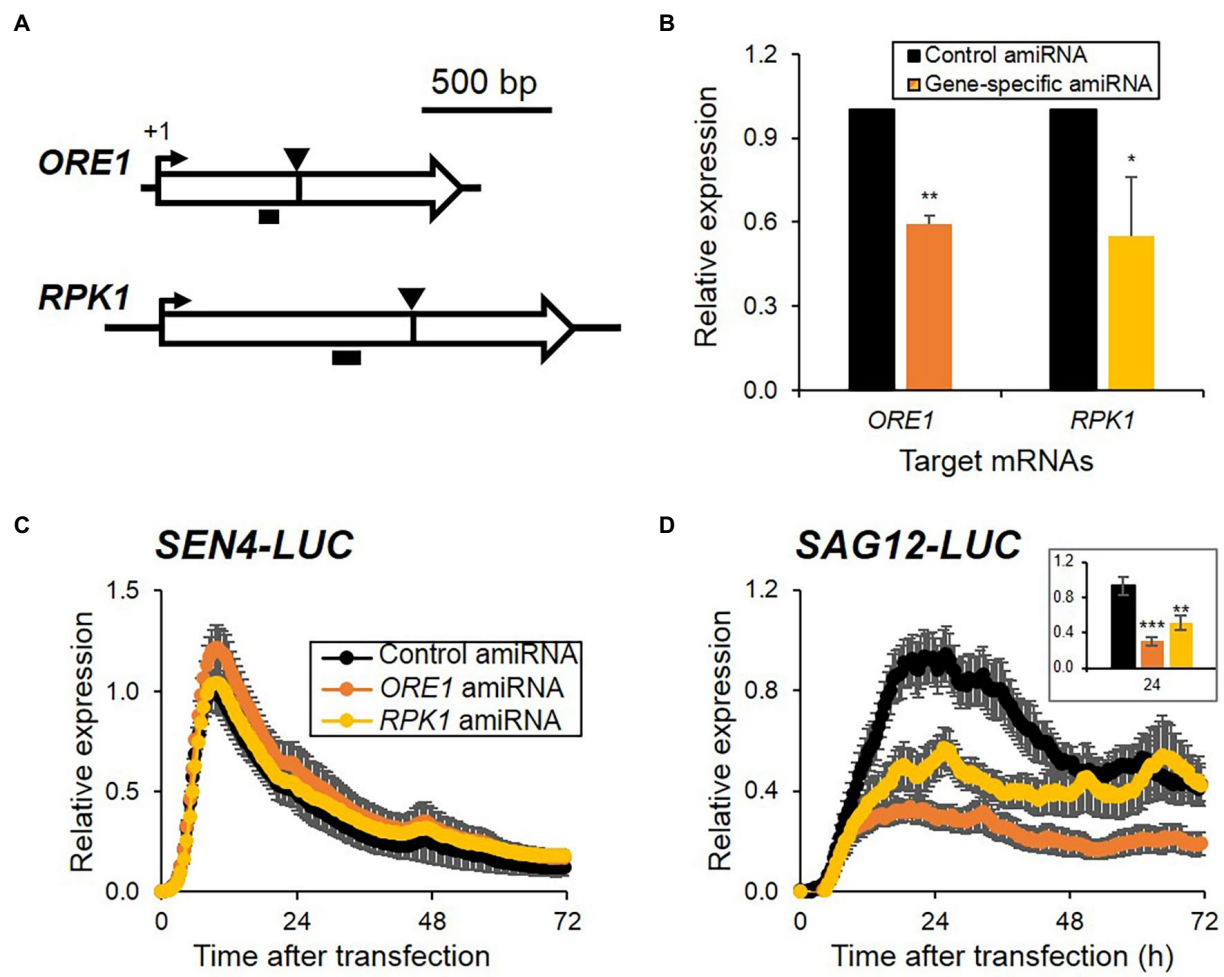
Expression patterns of *SEN4*- **(A)** and *SAG12-LUC* **(B)** reporters by reduced expression of senescence regulators using amiRNAs. **(A)** Schematic of the *ORE1* and *RPK1* with the amiRNA-targeted site (arrowheads) and the PCR amplified region (horizontal black bars) indicated for each. **(B)** Relative expression of *ORE1* and *RPK1* from protoplasts transfected with control or the gene-specific amiRNA indicated and harvested at 16 h incubation under dim light after transfection. Data represent mean ± SE (*n* = 3). **(C,D)** Bioluminescence traces of *SEN4*- **(C)** and *SAG12-LUC* **(D)** with amiRNA-construct gene targeting *ORE1* or *RPK1* during darkness. Symbols in **(D)** are the same as in **(C)**. (Inset) Bar graph of relative expression of *SAG12-LUC* at 24 h after transfection. Luciferase activity of LUC was normalized to the maximum level of RLUC over the assay. Relative expression of *SEN4*- **(C)** and *SAG12-LUC* **(D)** was calculated by normalization to the maximum level of LUC/RLUC throughout the measurement in protoplasts transfected with control amiRNA in each trial. Data represent mean ± SE (*n* = 6). A statistical analysis was performed using a two-tailed Student’s *t*-test (^*^p < 0.05; ^**^p < 0.01; and ^***^p < 0.001).

Artificial microRNA (amiRNA)-based knockdown approaches have been widely used for gene function studies in planta or protoplasts as a reverse-genetic approach (Ossowski et al., 2008; Kim and Somers, 2010). As an alternative and complementary approach to transient overexpression, we attempted to explore the feasibility of amiRNA-based knockdown approaches for investigating the functional regulatory role of genes in senescence regulation in protoplasts. We generated amiRNAs targeting *ORE1* and *RPK1* and validated their knockdown effect on endogenous target gene expression by qRT-PCR. *ORE1* and *RPK1* amiRNAs lowered the expression of each corresponding target gene by 41 and 45%, respectively, when compared with the control vector (Figures 3A,B). Because *RAV1* and *ORE7* are members of the Arabidopsis large family genes, and plants with loss-of-function or knockdown of *RAV1* and *ORE7* exhibited no senescence phenotypes (Lim et al., 2007b; Woo et al., 2010), the amiRNAs of *RAV1* and *ORE7* were excluded from a pilot test set of amiRNA-based knockdown approach. We examined the effects of *ORE1* and *RPK1* amiRNAs on the expression patterns of both *SEN4*- and *SAG12-LUC* reporters after co-transfection of effectors and reporters in protoplasts. *ORE1* and *RPK1* amiRNAs led to 2.7- and 1.6-fold reduction of *SAG12-LUC* expression at its peak time compared with control, respectively, although no effect on *SEN4-LUC* expression was observed (Figures 3C,D; Supplementary Table 2). As the reduction effect of *ORE1* and *RPK1* amiRNAs on *SAG12-LUC* is consistent with increased expression of *SAG12-LUC* by *ORE1* and *RPK1* overexpression (Figure 2), the amiRNAs approaches in protoplasts can be useful for assessing gene functions in senescence regulation. To further validate amiRNA approaches, we included additional amiRNA effectors of *ORE4* and *ORE9* whose loss-of-function mutants exhibited delayed leaf senescence, along with overexpression of *miR164B* (miR164B-OX), a senescence regulatory miRNA targeting *ORE1* (Woo et al., 2001, 2002; Kim et al., 2009). We failed to detect any significant change in *SEN4-LUC* when *ORE9*, *ORE4* amiRNAs, and miR164B-OX were introduced (Supplementary Figure 3A; Supplementary Table 2), which are similar when *ORE1* and *RPK1* amiRNAs were used. However, we observed a reduction in *SAG12-LUC* in protoplasts transfected with *ORE9* amiRNA and miR164B-OX, but no change in *SAG12-LUC* levels in protoplasts transfected with *ORE4* amiRNA (Supplementary Figure 3B; Supplementary Table 2). Since *ORE4* encodes plastid ribosomal small subunit protein 17, and the *ore4-1* mutant had no phenotype in dark-induced senescence, no change in *SAG12-LUC* by *ORE4* amiRNA can be explained. Collectively, these results indicate that the amiRNA-based knockdown approach with *SAG12-LUC* reporter is at least valid for a rapid functional assay of genes involved in senescence regulation, as is the overexpression approach.

### Divergent Function of ORE1 and RPK1 in Premature Cell Death Regulation Through Different ROS Signaling

**Figure 4:**
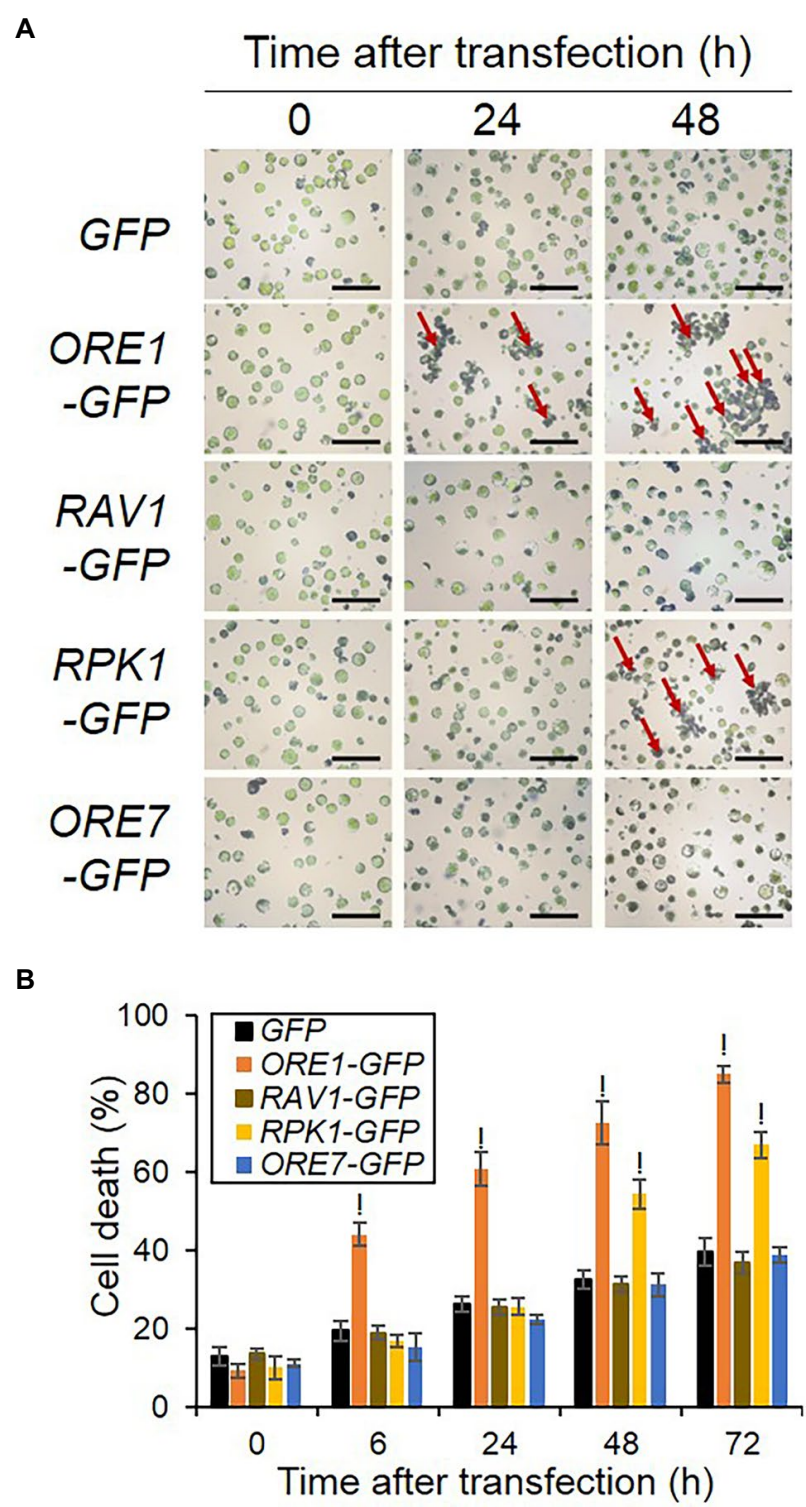
Kinetic cell death responses induced by overexpression of various senescence regulators in Arabidopsis protoplasts. **(A)** Representative images of protoplasts stained by Evans blue. **(B)** Time-series measurement of cell death in protoplasts expressing various senescence regulators. Protoplasts were transfected with empty *GFP* (Control), *ORE1-GFP*, or *RPK1-GFP* and stained with Evans blue dye at 0, 6, 24, 48, and 72 h post-transfection. Normal and stained cells were counted in five to 12 fields containing at least 50 cells from each sample under microscopy. The percentage of dead cells was measured as [stained cells/(stained + normal cells)] × 100%. Bars = 1 mm. Data represent mean ± SE (*n* = 3). A statistical analysis was performed using a two-tailed Student’s *t*-test (^!^*p* < 0.0001). Similar results were obtained in two independent trials.

Overexpression of selected senescence regulators led to different kinetic expression patterns of senescence reporters (Figure 2), with some of them, such as *ORE1* and *ORE7* inducing dampened expression. Since senescence accompanies death, cell death can induce suppressed or dampened *SEN4*- and *SAG12-LUC* expression in protoplasts. Therefore, we explored cell death responses in protoplasts where *ORE1*, *RAV1*, *RPK1*, and *ORE7* were overexpressed. We transfected plasmids of overexpression cassettes of *ORE1*, *RAV1*, *RPK1*, and *ORE7* in protoplasts and stained transfected protoplasts with Evans blue dyes in a time-dependent manner. We confirmed that the transfection efficiency in our assay was approximately 80% for all effectors, which was higher than 50% of the transfection efficiencies recommended for successful experiments (Supplementary Figure 4; Yoo et al., 2007). The extent of cell death accumulation in *ORE1*-overexpressing protoplasts significantly increased to 44.1% at 6 h and remained higher till to 85.0% at 72 h post-transfection, but that of control protoplasts was 19.5% at 6 h and 39.6% at 72 h post-transfection. Interestingly, *RPK1* overexpression at later incubation time points ranging from 48 to 72 h increased the cell death level to 67.0%, which is a similar level to that of *ORE1*. However, the expression of *RAV1* and *ORE7* did not affect cell death accumulation in the transfected protoplasts compared with that of control (Figure 4). We also confirmed that all effector proteins were expressed to a certain level up to 72 h, although ORE1 and RPK1 protein levels declined from 24 to 72 h, which was proposed to be due to the increasing proportion of dead cells induced by *ORE1* and *RPK1* overexpression (Supplementary Figure 5). These results indicate that the earlier induction of SAG promoters in *ORE1*-overexpressing protoplasts is due to premature cell death of protoplasts by *ORE1*. Furthermore, these results imply that *ORE1* and *RPK1* have different kinetic functions in triggering cell death during senescence.

**Figure 5:**
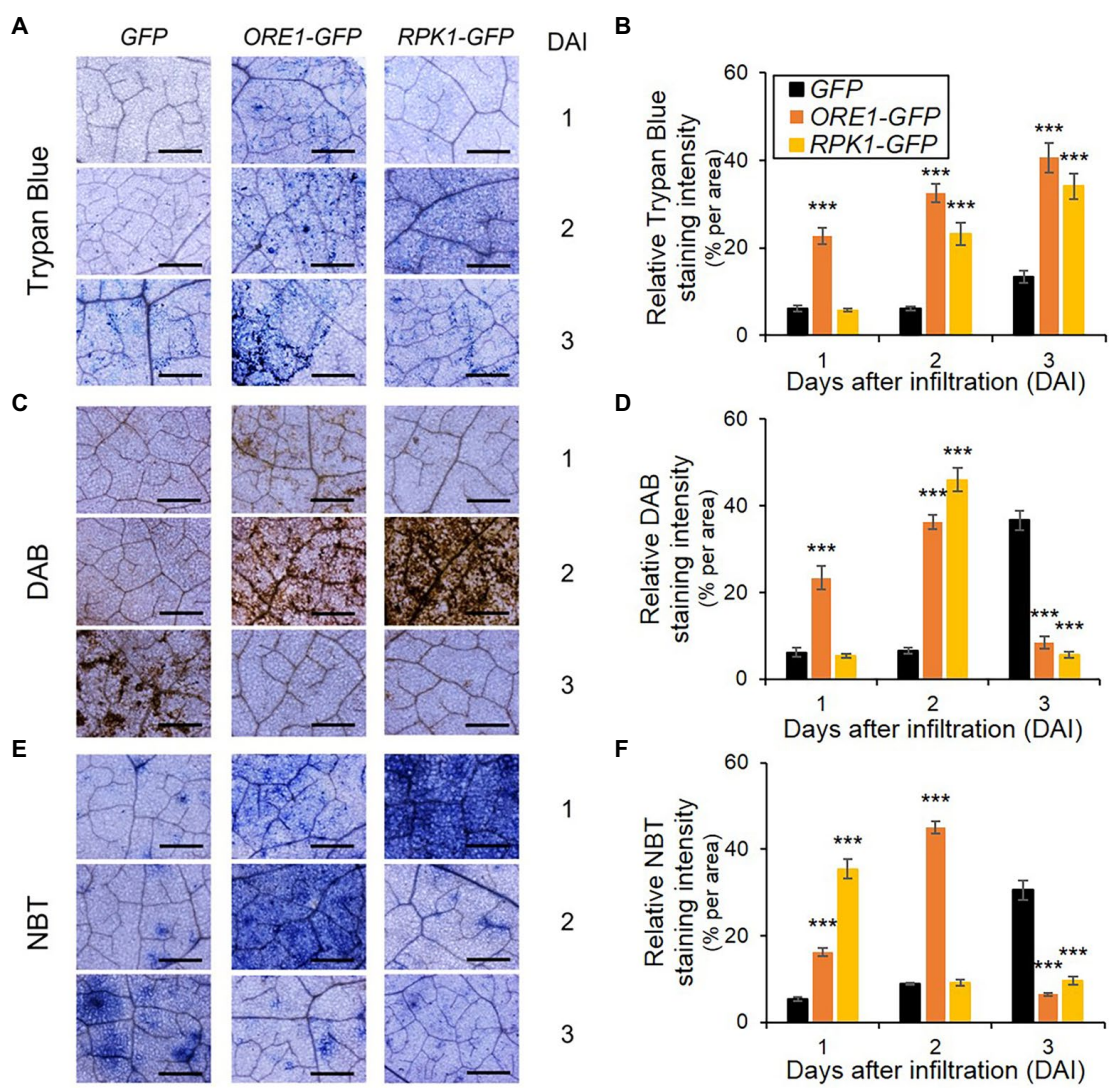
ROS accumulation and cell death responses in *Nicotiana benthamiana* leaves expressing senescence regulators. **(A,C,E)** Representative images of leaves stained using trypan blue **(A)**, DAB **(C)**, and NBT **(E)** at indicated days after infiltration (DAI). *GFP* (Control), *ORE1-GFP*, or *RPK1-GFP* were transiently overexpressed in four-week-old *N. benthamiana* leaves through Agrobacteria infiltration. Subsequently, staining was conducted using their leaves on indicated DAI. Bars = 100 mm. **(B,D,F)** Time-series quantification of staining intensity from trypan blue **(B)**, DAB **(D)**, and NBT **(F)** images. Data represent mean ± SE (*n* = 5). Statistical analysis was conducted using a two-tailed Student’s *t*-test (^***^p < 0.001), and similar results were obtained in three independent trials.

*RPK1* and *ORE1* mediate ROS signaling and/or production to trigger age-dependent cell death (Balazadeh et al., 2010; Koo et al., 2017). Therefore, we dissected the accumulation rate of two major ROS species, H_2_O_2,_ and superoxide, as well as cell death in tobacco tissues that ectopically expressed *RPK1* and *ORE1* (Figure 5). Cell death, H_2_O_2_, and superoxide were visualized using trypan blue, DAB, and NBT, respectively. Trypan blue-mediated cell death assay in tobacco exhibited similar results as shown in protoplasts: *ORE1* overexpression provoked an earlier onset of cell death marked with blue stains in tobacco leaves from 1 day after infiltration (DAI), whereas *RPK1* and *GFP* control induced detectable cell death at 2 DAI and 3 DAI, respectively (Figure 5A). Similarly, DAB-mediated H_2_O_2_ detection with brown staining revealed earlier and higher accumulation of H_2_O_2_ at 1 DAI in *ORE1*-expressed leaves only, and 2 DAI in both *ORE1*- and *RPK1*-expressed leaves, compared with those of GFP-expressed leaves (Figure 5B). However, superoxide staining with NBT produced a higher level of blue staining in leaves expressing *RPK1* than *ORE1* in 1 DAI, although *ORE1*-expressed leaves had higher stains than those of control at 2 DAI (Figure 5C). Collectively, these results indicate that *ORE1* and *RPK1* might be involved in acute cell death and superoxide-dependent intermediate cell death during leaf senescence, respectively.

## Discussion

Leaf senescence is the final developmental phase with self-disposal, yet it is essential for energy recycling in other organs. Although plants coordinate their leaf development, leaf senescence begins with cell-autonomous determination by multiple genetic factors (Thomas et al., 2003). The protoplast-based transient expression system is a cell-based functional assay technique that is efficient and adaptable for studying various plant developmental and physiological responses (Yoo et al., 2007; Lehmann et al., 2020). In this study, we demonstrated the potential utility of a transient gene expression system using Arabidopsis mesophyll protoplasts to assess the functional role of genes in senescence regulation in plants. We used two *SEN4*- and *SAG12-LUC* reporters for kinetic senescence response assay and validated the feasibility of functional assessment of genes in senescence regulation using their overexpression and knockdown approaches (Figures 2, 3). Furthermore, we investigated the functional difference between *ORE1* and *RPK1* in cell death-mediated senescence by combining the kinetic responses of the reporter assay and histochemical assay (Figures 2, 4, 5).

### Evaluation of Senescence Response Assay Using Arabidopsis Mesophyll Protoplasts

To dissect senescence responses in protoplasts, we established two reporter sets of *SEN4*- and *SAG12-LUC* for analyzing early and late kinetic responsiveness (Figure 1). *SEN4* and *SAG12* were identified as transcriptionally upregulated SAGs with different kinetic profiles and have been used as molecular markers for senescence responses (Noh and Amasino, 1999; Woo et al., 2002). However, the expression pattern of *SEN4-LUC* and *SAG12-LUC* reporters in transfected protoplasts exhibited rapid induction followed by a decline, which is different from that observed in intact plants. These patterns could be attributed to the strong basal expression of many transfected plasmids and the reduction of reporter expression due to accumulating cell death over the assay. Also, we tested the possible usage of other downregulated SAGs as potential reporters, but their expression levels were marginal or variable in the protoplasts (Supplementary Figure 1). Nonetheless, we validated a potential usage of *SEN4*- and *SAG12-LUC* reporters for senescence responses in the protoplast system. Overexpression of identified positive senescence regulators, including *ORE1*, *RAV1*, and *RPK1*, as well as negative senescence regulators, including *ORE7*, affected the expression level of both *SEN4-LUC* and *SAG12-LUC* consistently with reports using intact leaves (Figure 2; Lim et al., 2007b; Kim et al., 2009; Woo et al., 2010; Lee et al., 2011). Furthermore, knockdown approaches based on amiRNA technology can be used for protoplast-based senescence assay using *SAG12-LUC*. The introduction of amiRNA constructs targeting *ORE1*, *RPK1*, or *ORE9*, as well as miR164B-OX construct, together with *SAG12-LUC* resulted in reduced *SAG12* expression in the protoplasts (Figure 3; Supplementary Figure 3). These results reinforce the notion that leaf senescence occurs by cell-autonomous signals in the mature stage. However, this does not exclude the possible involvement of an early developmental signal or additional intercellular communication in senescence regulation. This might be a reason why amiRNA for *ORE4* encoding plastid ribosomal proteins failed to show any effects on *SAG12* expression. Another limitation of this approach might be the weak or marginal responsiveness of *SEN4-LUC* reporter different from that of *SAG12-LUC* when amiRNA constructs are used as effectors. It is proposed to be because the expression of transfected *SEN4-LUC* was quickly induced up to a certain level before the substantial removal of endogenous target genes by transfected amiRNAs. Alternatively, *SEN4-LUC* can be less responsive to the repression effect by anti-senescence signals before or at the beginning of senescence since a basal expression of *SEN4* should be maintained for cell elongation in leaf growth (Woo et al., 2001; Lee et al., 2018). This is also supported by a lower effect of *ORE7* overexpression in *SEN4-LUC* than *SAG12-LUC* (Figures 2A,B). We also noticed a potential weakness of this approach in that cell death can cause dampened or suppressed luciferase activity, which misleads the impact of transfected effectors (Figure 4). Nevertheless, protoplast-based senescence assays can be used for studies of leaf senescence by complementing molecular genetic approaches based on Arabidopsis mutants or transgenic plants. As transient transfection in protoplasts can deliver multiple plasmids simultaneously, the function of multifamily genes or interaction between senescence regulators can be dissected rapidly before laborious genetic approaches. The function or effectiveness of genes in senescence responses can be analyzed or compared in differentiated and defined protoplasts extracted from normal mature leaves, which can avoid a potential misleading interpretation by indirect or malfunctional effects of genes in an early development stage. Additionally, the protoplast-based senescence assay can be applied to high-throughput analysis based on the use of a genome-wide collection of amiRNA or open reading frame (ORF) clones, or chemical libraries.

An advantage of using this system was exemplified by comparing the functional effectiveness of known senescence regulatory genes based on the timing-dependent responses of reporters. The temporal expression patterns of SAGs can reveal the timing of gene involvement from initiation to termination of senescence. Among positive senescence regulators, *ORE1* overexpression resulted in the earliest induction of both reporters at post-transfection (Figures 2C,D). This is consistent with the results using amiRNA approaches for *ORE1* and *RPK1* (Figure 3D). This indicates *ORE1* could function as a primary and crucial genetic factor for senescence initiation. This is consistent with the role of *ORE1* as the primary genetic factor in the death circuit with the trifurcate feed-forward pathway involving EIN2, *ORE1*, and miR164 (Kim et al., 2009). Interestingly, the effectiveness of *RAV1* on the expression of reporters appears later compared with other senescence regulators. Although a previous study suggested that *RAV1* is a transcription factor with a role in triggering the initiation of leaf senescence (Woo et al., 2010), it may function as an intermediate factor following the action of primary factors like ORE1. The protoplast kinetic approach could give a more informative clue in uncovering *in vivo* role of genes over the traditional phenotypic evaluation approach. Another analytic window of reporter responses is their expression pattern. *ORE7* overexpression led to completely dampened expression (*SAG12-LUC*) and shortly induced, but dampened expression (*SEN4-LUC*; Figures 2A,B). It is consistent with a previous report that AT-hook protein *ORE7* functions as an epigenetic regulator for leaf senescence (Lim et al., 2007b). *ORE7* might induce chromatin condensation, which blocks the transcriptional activation of *SAG12-LUC* completely, and later induction of *SEN4-LUC*. Intriguingly, *ORE1* overexpression also dampened expression of *SEN4-LUC* and *SAG-LUC*, but it followed a higher induction of both reporters at early time points (Figures 2A,B). This implied that *ORE1* has a different molecular function in the regulation of senescence from the *ORE7*-mediated repression of SAGs.

### *ORE1* and *RPK1* in the Regulation of Cell Death-Mediated Senescence

Senescence involves the gradual loss of cellular activity and ends with death. However, an increasing amount of evidence suggests that cell death processes are not only required for dismantlement and relocation of cellular macromolecules during senescence but also mediate the initiation of leaf senescence (Guiboileau et al., 2010). An advantage of using protoplasts is the easy application to envision investigating cellular biological phenotypes combined using fluorescence-based reporters or exogenous staining. Dampened levels in the luciferase-based readout can appear not only due to strong repression but also due to cell death. Therefore, we used Evans blue staining for investigating cell death responses as senescence. *ORE1* and *RPK1* overexpression enhanced the extent of cell death, although *ORE1* increased the extent of cell death much earlier time points than *RPK1* did (Figure 4). Interestingly, *ORE7* had little effect on the extent of cell death, indicating the dampened expression of reporters is due to the epigenetic repression of *ORE7*. Contrarily, dampened expression of reporters by *ORE1* is likely due to the early cell death of protoplasts. Earlier and rapid induction of *SEN4* and *SAG12-LUC* by ORE1 supported the early onset of cell death signals (Figure 2). Additionally, *RPK1* overexpression showed enhanced cell death at later incubation time points compared with *ORE1*. Early provocation of cell death by *ORE1* supports the notion that cell death signals are likely to trigger senescence responses. Furthermore, our results suggest that *ORE1* and *RPK1* share a convergent pathway leading to senescence and cell death, but through different intermediate regulatory signaling. H_2_O_2_ and superoxide signaling are likely involved in *ORE1* and *RPK1*-mediated cell death and senescence (Figure 5). Cell death induced by *ORE1* was observed at the same time with the accumulation of H_2_O_2_ and superoxide, but *RPK1*-mediated cell death along with H_2_O_2_ production followed a rapid accumulation of superoxide. It is unclear whether ROS-induced by *ORE1* is a result or cause of cell death. In the first scenario, these results indicate that *ORE1* regulates cell death directly but *RPK1* does it indirectly through superoxide. These results are consistent with previous reports: ORE1 regulates aging-induced cell death and senescence (Kim et al., 2009); RPK1, CaM4, and RbohF Trio regulate age-dependent cell death *via* the accumulation of the superoxide (Koo et al., 2017). Alternatively, given that ROS is one of the critical factors triggering cell death, *ORE1* may also be involved in ROS-mediated senescence similar to *RPK1*. However, there is no clear evidence on the interaction between *ORE1*- and *RPK1*-mediated pathways in senescence regulation. Future works will seek to resolve the molecular mechanisms underpinning the interaction between *ORE1*- and *RPK1*-mediated pathways in cell death, including the potential involvement of ROS.

### Senescence Regulatory Scheme

Based on our study, we suggest a regulatory and kinetic scheme of cellular senescence program regarding how senescence regulatory genes, such as *ORE1*, *RPK1*, *RAV1*, and *ORE7*, are involved in senescence regulation, reflected by the expression of *SEN4* and *SAG12*, partially through cell death and ROS-mediated signaling (Supplementary Figure 6). *ORE1* and *RPK1* function as early positive senescence regulators through cell death- and ROS-induced senescence, respectively. *RAV1* might be involved in senescence responses as a late positive senescence regulator through the different pathways from *ORE1* and *RPK1* signals. *ORE7* is an epigenetic negative regulator that plays a dual-temporal role in the regulation of SAG expression. This scheme can provide novel insights for temporal regulatory involvement of senescence genes, although mechanistic relationships among the senescence regulators are not clearly defined in this scheme. Future studies with more diverse senescence regulators under various senescence triggering conditions will reveal a more reliable and clearer map for a kinetic function of senescence regulators during leaf senescence.

Overall, our results indicate that the protoplast transient expression system based on the luciferase-based assay is an effective tool for rapid functional dissection of senescence regulators in Arabidopsis. Combining other cellular reporters or different protoplast sources will enable us to broaden the utility of our approaches for studying various senescence processes in Arabidopsis, as well as other non-model plants.

## Data Availability Statement

The raw data supporting the conclusions of this article will be made available by the authors, without undue reservation.

## Author Contributions

PPTD and JK conceived and designed the experiments and wrote the paper. PPTD, JHK, and JK performed the experiments and analyzed the data. All authors contributed to the article and approved the submitted version.

## Funding

This work was supported by the research grant of Jeju National University in 2020.

## Conflict of Interest

The authors declare that the research was conducted in the absence of any commercial or financial relationships that could be construed as a potential conflict of interest.

## Publisher’s Note

All claims expressed in this article are solely those of the authors and do not necessarily represent those of their affiliated organizations, or those of the publisher, the editors and the reviewers. Any product that may be evaluated in this article, or claim that may be made by its manufacturer, is not guaranteed or endorsed by the publisher.

## Footnotes

^1^ http://wmd3.weigelworld.org

^2^ https://imagej.nih.gov/ij/

## References

[ref1] Ambastha V. Sopory S. K. Tiwari B. S. Tripathy B. C. (2017). Photo-modulation of programmed cell death in rice leaves triggered by salinity. Apoptosis 22, 41–56. doi: 10.1007/s10495-016-1305-7, PMID: 27747443

[ref2] Balazadeh S. Wu A. Mueller-Roeber B. (2010). Salt-triggered expression of the ANAC092-dependent senescence regulon in *Arabidopsis thaliana*. Plant Signal. Behav. 5, 733–735. doi: 10.4161/psb.5.6.11694, PMID: 20404534 PMC3001574

[ref3] Camargo Rodriguez A. V. (2021). Integrative Modelling of gene expression and digital phenotypes to describe senescence in wheat. Genes 12:909. doi: 10.3390/genes12060909, PMID: 34208213 PMC8230903

[ref4] Domozych D. S. Ritter E. Lietz A. Tinaz B. Raimundo S. C. (2020). Protoplast isolation and manipulation in the unicellular model plant Penium margaritaceum. Methods Mol. Biol. 2149, 111–124. doi: 10.1007/978-1-0716-0621-6_7, PMID: 32617932

[ref5] Guiboileau A. Sormani R. Meyer C. Masclaux-Daubresse C. (2010). Senescence and death of plant organs: nutrient recycling and developmental regulation. C. R. Biol. 333, 382–391. doi: 10.1016/j.crvi.2010.01.016, PMID: 20371113

[ref6] Have M. Marmagne A. Chardon F. Masclaux-Daubresse C. (2017). Nitrogen remobilization during leaf senescence: lessons from Arabidopsis to crops. J. Exp. Bot. 68, 2513–2529. doi: 10.1093/jxb/erw365, PMID: 27707774

[ref7] Hickman R. Hill C. Penfold C. A. Breeze E. Bowden L. Moore J. D.. (2013). A local regulatory network around three NAC transcription factors in stress responses and senescence in Arabidopsis leaves. Plant J. 75, 26–39. doi: 10.1111/tpj.12194, PMID: 23578292 PMC3781708

[ref8] Hwang I. Sheen J. (2001). Two-component circuitry in Arabidopsis cytokinin signal transduction. Nature 413, 383–389. doi: 10.1038/35096500, PMID: 11574878

[ref9] Juszczak I. Baier M. (2014). Quantification of superoxide and hydrogen peroxide in leaves. Methods Mol. Biol. 1166, 217–224. doi: 10.1007/978-1-4939-0844-8_16, PMID: 24852638

[ref10] Kim H. J. Hong S. H. Kim Y. W. Lee I. H. Jun J. H. Phee B. K.. (2014). Gene regulatory cascade of senescence-associated NAC transcription factors activated by ETHYLENE-INSENSITIVE2-mediated leaf senescence signalling in Arabidopsis. J. Exp. Bot. 65, 4023–4036. doi: 10.1093/jxb/eru112, PMID: 24659488 PMC4106440

[ref11] Kim J. Kim J. H. Lyu J. I. Woo H. R. Lim P. O. (2018b). New insights into the regulation of leaf senescence in Arabidopsis. J. Exp. Bot. 69, 787–799. doi: 10.1093/jxb/erx287, PMID: 28992051

[ref12] Kim J. Kim Y. Yeom M. Kim J. H. Nam H. G. (2008). FIONA1 is essential for regulating period length in the Arabidopsis circadian clock. Plant Cell 20, 307–319. doi: 10.1105/tpc.107.055715, PMID: 18281507 PMC2276451

[ref13] Kim H. J. Park J. H. Kim J. Kim J. J. Hong S. Kim J.. (2018a). Time-evolving genetic networks reveal a NAC troika that negatively regulates leaf senescence in Arabidopsis. Proc. Natl. Acad. Sci. U. S. A. 115, E4930–E4939. doi: 10.1073/pnas.1721523115, PMID: 29735710 PMC6003463

[ref14] Kim J. Park S. J. Lee I. H. Chu H. Penfold C. A. Kim J. H.. (2018c). Comparative transcriptome analysis in Arabidopsis ein2/ore3 and ahk3/ore12 mutants during dark-induced leaf senescence. J. Exp. Bot. 69, 3023–3036. doi: 10.1093/jxb/ery137, PMID: 29648620 PMC5972659

[ref15] Kim J. Somers D. E. (2010). Rapid assessment of gene function in the circadian clock using artificial microRNA in Arabidopsis mesophyll protoplasts. Plant Physiol. 154, 611–621. doi: 10.1104/pp.110.162271, PMID: 20709829 PMC2949038

[ref16] Kim J. H. Woo H. R. Kim J. Lim P. O. Lee I. C. Choi S. H.. (2009). Trifurcate feed-forward regulation of age-dependent cell death involving miR164 in Arabidopsis. Science 323, 1053–1057. doi: 10.1126/science.1166386, PMID: 19229035

[ref17] Koo J. C. Lee I. C. Dai C. Lee Y. Cho H. K. Kim Y.. (2017). The protein trio RPK1-CaM4-RbohF mediates transient superoxide production to trigger age-dependent cell death in Arabidopsis. Cell Rep. 21, 3373–3380. doi: 10.1016/j.celrep.2017.11.077, PMID: 29262318

[ref18] Koyama T. (2018). A hidden link between leaf development and senescence. Plant Sci. 276, 105–110. doi: 10.1016/j.plantsci.2018.08.006, PMID: 30348308

[ref19] Lee I. C. Hong S. W. Whang S. S. Lim P. O. Nam H. G. Koo J. C. (2011). Age-dependent action of an ABA-inducible receptor kinase, RPK1, as a positive regulator of senescence in Arabidopsis leaves. Plant Cell Physiol. 52, 651–662. doi: 10.1093/pcp/pcr026, PMID: 21382977

[ref20] Lee Y. K. Rhee J. Y. Lee S. H. Chung G. C. Park S. J. Segami S.. (2018). Functionally redundant LNG3 and LNG4 genes regulate turgor-driven polar cell elongation through activation of XTH17 and XTH24. Plant Mol. Biol. 97, 23–36. doi: 10.1007/s11103-018-0722-0, PMID: 29616436

[ref21] Lehmann S. Dominguez-Ferreras A. Huang W. J. Denby K. Ntoukakis V. Schafer P. (2020). Novel markers for high-throughput protoplast-based analyses of phytohormone signaling. PLoS One 15:e0234154. doi: 10.1371/journal.pone.0234154, PMID: 32497144 PMC7272087

[ref22] Li N. Uhrig J. F. Thurow C. Huang L. J. Gatz C. (2019). Reconstitution of the Jasmonate signaling pathway in plant protoplasts. Cell 8:532. doi: 10.3390/cells8121532, PMID: 31795159 PMC6953042

[ref23] Li Z. Woo H. R. Guo H. (2018). Genetic redundancy of senescence-associated transcription factors in Arabidopsis. J. Exp. Bot. 69, 811–823. doi: 10.1093/jxb/erx345, PMID: 29309664

[ref24] Lim P. O. Kim Y. Breeze E. Koo J. C. Woo H. R. Ryu J. S.. (2007b). Overexpression of a chromatin architecture-controlling AT-hook protein extends leaf longevity and increases the post-harvest storage life of plants. Plant J. 52, 1140–1153. doi: 10.1111/j.1365-313X.2007.03317.x, PMID: 17971039

[ref25] Lim P. O. Kim H. J. Nam H. G. (2007a). Leaf senescence. Annu. Rev. Plant Biol. 58, 115–136. doi: 10.1146/annurev.arplant.57.032905.105316 17177638

[ref26] Lim P. O. Woo H. R. Nam H. G. (2003). Molecular genetics of leaf senescence in Arabidopsis. Trends Plant Sci. 8, 272–278. doi: 10.1016/S1360-1385(03)00103-1 12818661

[ref27] Noh Y. S. Amasino R. M. (1999). Identification of a promoter region responsible for the senescence-specific expression of SAG12. Plant Mol. Biol. 41, 181–194. doi: 10.1023/a:1006342412688, PMID: 10579486

[ref28] Ossowski S. Schwab R. Weigel D. (2008). Gene silencing in plants using artificial microRNAs and other small RNAs. Plant J. 53, 674–690. doi: 10.1111/j.1365-313X.2007.03328.x, PMID: 18269576

[ref29] Rolland V. (2018). Determining the subcellular localization of fluorescently tagged proteins using protoplasts extracted from transiently transformed *Nicotiana benthamiana* leaves. Methods Mol. Biol. 1770, 263–283. doi: 10.1007/978-1-4939-7786-4_16, PMID: 29978408

[ref30] Schwab R. Ossowski S. Riester M. Warthmann N. Weigel D. (2006). Highly specific gene silencing by artificial microRNAs in Arabidopsis. Plant Cell 18, 1121–1133. doi: 10.1105/tpc.105.039834, PMID: 16531494 PMC1456875

[ref31] Stigter K. A. Plaxton W. C. (2015). Molecular mechanisms of phosphorus metabolism and transport during leaf senescence. Plants 4, 773–798. doi: 10.3390/plants4040773, PMID: 27135351 PMC4844268

[ref32] Thomas H. Ougham H. J. Wagstaff C. Stead A. D. (2003). Defining senescence and death. J. Exp. Bot. 54, 1127–1132. doi: 10.1093/jxb/erg133 12654863

[ref33] Tyurin A. A. Suhorukova A. V. Kabardaeva K. V. Goldenkova-Pavlova I. V. (2020). Transient gene expression is an effective experimental tool for the research into the fine mechanisms of plant gene function: advantages, limitations, and solutions. Plan. Theory 9:1187. doi: 10.3390/plants9091187, PMID: 32933006 PMC7569937

[ref34] Untergasser A. Cutcutache I. Koressaar T. Ye J. Faircloth B. C. Remm M.. (2012). Primer3--new capabilities and interfaces. Nucleic Acids Res. 40:e115. doi: 10.1093/nar/gks596, PMID: 22730293 PMC3424584

[ref35] Vachova H. Alquicer G. Sedinova M. Sachova J. Hradilova M. Varga V. (2020). A rapid approach for in locus overexpression of Trypanosoma brucei proteins. Mol. Biochem. Parasitol. 239:111300. doi: 10.1016/j.molbiopara.2020.111300, PMID: 32682799

[ref36] Voinnet O. Rivas S. Mestre P. Baulcombe D. (2003). An enhanced transient expression system in plants based on suppression of gene silencing by the p19 protein of tomato bushy stunt virus. Plant J. 33, 949–956. doi: 10.1046/j.1365-313x.2003.01676.x, PMID: 12609035

[ref37] Woo H. R. Chung K. M. Park J. H. Oh S. A. Ahn T. Hong S. H.. (2001). ORE9, an F-box protein that regulates leaf senescence in Arabidopsis. Plant Cell 13, 1779–1790. doi: 10.1105/TPC.010061, PMID: 11487692 PMC139127

[ref38] Woo H. R. Goh C. H. Park J. H. Teyssendier de la Serve B. Kim J. H. Park Y. I.. (2002). Extended leaf longevity in the ore4-1 mutant of Arabidopsis with a reduced expression of a plastid ribosomal protein gene. Plant J. 31, 331–340. doi: 10.1046/j.1365-313x.2002.01355.x, PMID: 12164812

[ref39] Woo H. R. Kim J. H. Kim J. Kim J. Lee U. Song I. J.. (2010). The RAV1 transcription factor positively regulates leaf senescence in Arabidopsis. J. Exp. Bot. 61, 3947–3957. doi: 10.1093/jxb/erq206, PMID: 20826506 PMC2935868

[ref40] Woo H. R. Kim H. J. Lim P. O. Nam H. G. (2019). Leaf senescence: systems and dynamics aspects. Annu. Rev. Plant Biol. 70, 347–376. doi: 10.1146/annurev-arplant-050718-095859, PMID: 30811218

[ref41] Woo H. R. Koo H. J. Kim J. Jeong H. Yang J. O. Lee I. H.. (2016). Programming of plant leaf senescence with temporal and inter-Organellar coordination of Transcriptome in Arabidopsis. Plant Physiol. 171, 452–467. doi: 10.1104/pp.15.01929, PMID: 26966169 PMC4854694

[ref42] Yoo S. D. Cho Y. H. Sheen J. (2007). Arabidopsis mesophyll protoplasts: a versatile cell system for transient gene expression analysis. Nat. Protoc. 2, 1565–1572. doi: 10.1038/nprot.2007.199, PMID: 17585298

[ref43] Yu G. Wang X. Chen Q. Cui N. Yu Y. Fan H. (2019). Cucumber mildew resistance locus O interacts with Calmodulin and regulates plant cell death associated with plant immunity. Int. J. Mol. Sci. 20:995. doi: 10.3390/ijms20122995, PMID: 31248151 PMC6627319

[ref44] Zhang D. Zhang N. Shen W. Li J. F. (2019). Engineered artificial MicroRNA precursors facilitate cloning and gene silencing in Arabidopsis and Rice. Int. J. Mol. Sci. 20:620. doi: 10.3390/ijms20225620, PMID: 31717686 PMC6888491

