# Supplementary material for: Rapid Investigation of Functional Roles of Genes in Regulation of Leaf Senescence Using Arabidopsis Protoplasts
Source: Front Plant Sci. 2022 Mar 17;13:818239. doi: 10.3389/fpls.2022.818239 (PMC8969776; doi:10.3389/fpls.2022.818239)
Supplement: Supplementary file 1 [file Data_Sheet_1.docx]

SUPPLEMENTAL INFORMATION

Rapid Investigation of Functional Roles of Genes in Regulation of Leaf Senescence Using Arabidopsis Protoplasts

Phan Phuong Thao Doan^1^, Jin Hee Kim^2^, Jeongsik Kim^1,2,3*^

^1^Interdisciplinary Graduate Program in Advanced Convergence Technology & Science, Jeju National University, Jeju, South Korea, ^2^Subtropical Horticulture Research Institute, Jeju National University, Jeju, South Korea, ^3^Faculty of Science Education, Jeju National University, Jeju, South Korea

Email: yorus@jejunu.ac.kr (J. Kim)

**This file includes:**

Supplementary Materials and Methods

Supplementary Tables 1, 2, and 3

Supplementary Figures 1, 2, 3, 4, 5, and 6

Supplementary Reference

# Supplementary Materials and Methods

**Plasmid Construction**

We generated plasmid constructs for transient gene expression in protoplasts using GATEWAY cloning technology (Invitrogen, United States). For the reporter plasmids (*CA1*-, *THIONIN*-, and *PRK*-*LUC*), the 5′ upstream regions encompassing the 35S, *CA1* (At3g01500), *THIONIN* (At1g66100), and *PRK* (At1g32060) promoters were amplified by polymerase chain reaction (PCR) with Pfu DNA polymerase, using Arabidopsis Col-0 genomic DNA as a template and the following sets of primers (**Supplementary Table 1**). We subcloned the amplified DNA fragments into the entry vector of pCR-CCD-F using the corresponding restriction enzymes to produce entry clones. The promoter-LUC final constructs were established by LR recombination using the corresponding entry clones and gateway version of pOmegaLUC_SK+ vector (Kim and Somers, 2010). 35S-LUC plasmid and empty vector of pOmegaLUC_SK+ were used as negative controls for pattern and level of expression. For amiRNA effectors, ORE4 and ORE9 amiRNA plasmids were generated by digesting pAmiR-ORE4 (CSHL_027820) and pAmiR-ORE9 (CSHL_038083) with *Pst*I and *Bam*HI, then, ligating each resulting amiRNA foldback fragment into *Pst*I/*Bam*HI digested pCsVMV-PP2C-AmiR vector (Schwab et al., 2006; Kim and Somers, 2010). For miR164B-OX effector, the full-length expression cassette of miR164B was amplified using PCR from Arabidopsis Col-0 genomic DNA with Pfu DNA polymerase and gene-specific primers (**Supplementary Table 1**). Further, we subcloned the amplified DNA fragment into the entry vector of pCR-CCD-F using the corresponding restriction enzymes to produce entry clone. Then, we recombined the entry clone using gateway version of pCsVMV-N-999 to produce the effector plasmid of miR164B-OX.

## Luminescence Measurement

The luminescence measurement was performed as described in the Luminescence Measurement in Materials and Methods section, but normalized expression was used as the value of luciferase activity. Normalized expression was calculated using the formula:

$$\mathbf{Normalized expression}\boldsymbol{=}{\frac{\mathbf{LUC} \mathbf{luminescence}}{\mathbf{max (RLUC} \mathbf{luminescence)}}\boldsymbol{*100}}$$

## Subcellular Localization in protoplasts

We determined the subcellular localization and transfection efficiency of GFP, ORE1-GFP, RAV1-GFP, RPK1-GFP, and ORE7-GFP in protoplasts after a 18h transfection. GFP fluorescence was observed under an epifluorescence microscope (Axioscope A1, Carl Zeiss, Germany) and a BP505-530 filter. The transformation efficiency was measured as a bright green fluorescent protoplast number in view/total protoplast number in view (%). At least five photographs were taken for each sample, and these experiments were independently conducted at least twice. We processed and pseudocolored images using Photo Pos Pro 3 (Power of Software) or Photoshop 2018 (Adobe Systems). Red chlorophyll fluorescence was used to indicate the intercellular location of chloroplasts.

**Immunoblot analysis**

Protein abundance of effectors in protoplasts was assayed by Immunoblot analysis. We harvested and extracted protein from transfected protoplasts at different time points (6, 24, 48, and 72h after transfection) using 50μl protein extraction buffer (50mm Tris–HCl [pH 7.5], 150mm NaCl, 0.5% Nonidet P-40, 1mm EDTA, 1mm dithiothreitol, 1mm phenylmethylsulfonyl fluoride, 1 × complete protease inhibitor cocktail™ (Roche, Switzerland), and 50μm MG132). 15μl total protein was separated using SDS-PAGE and transferred to the Immobilon PVDF membrane (Millipore, Sigma, United States). Immunodetection was performed using an anti-GFP antibody (1:3000, ab6556, Abcam, United Kingdom) or anti-actin antibody (1:3000, A0480, Sigma, United States) and visualized using SuperSignal West Pico (Thermo Fisher Scientific Inc., United States) through an ImageQuant LAS4000 mini analyzer (GE Healthcare, United States).

# Supplementary Figures and Tables

## Supplementary Figures


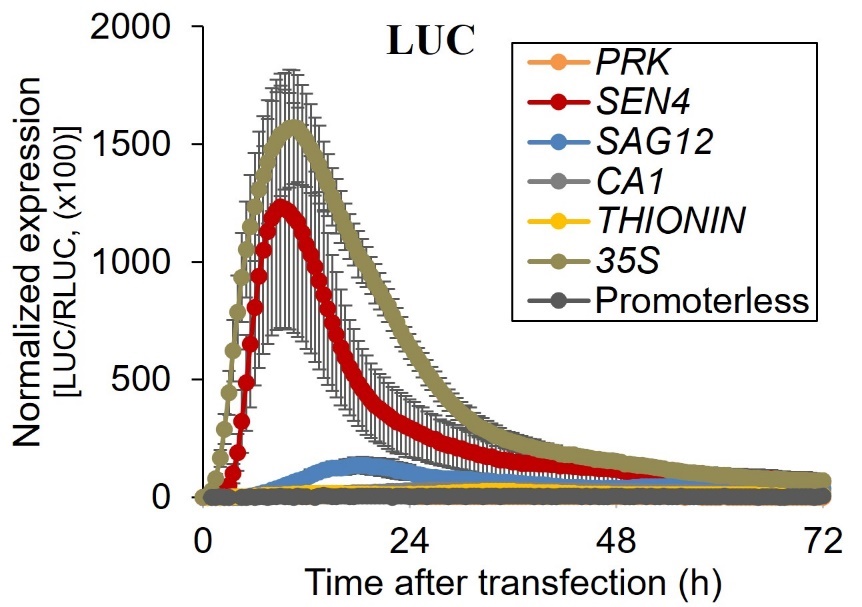


**Supplementary Figure 1.** Bioluminescence expression patterns of luciferase reporters under the control of other senescence-associated genes. Transfection and imaging acquisition were performed, as in **Figure 1** with *SEN4-*, *SAG12-*, *CA1-*, *THIONIN-*, *PRK-, 35S-,* or Promoterless-LUC as a reporter. Each data set was normalized to the maximum level of RLUC throughout the measurement. Data represent mean ± SE (*n* = 3). Similar results were obtained in two independent trials.


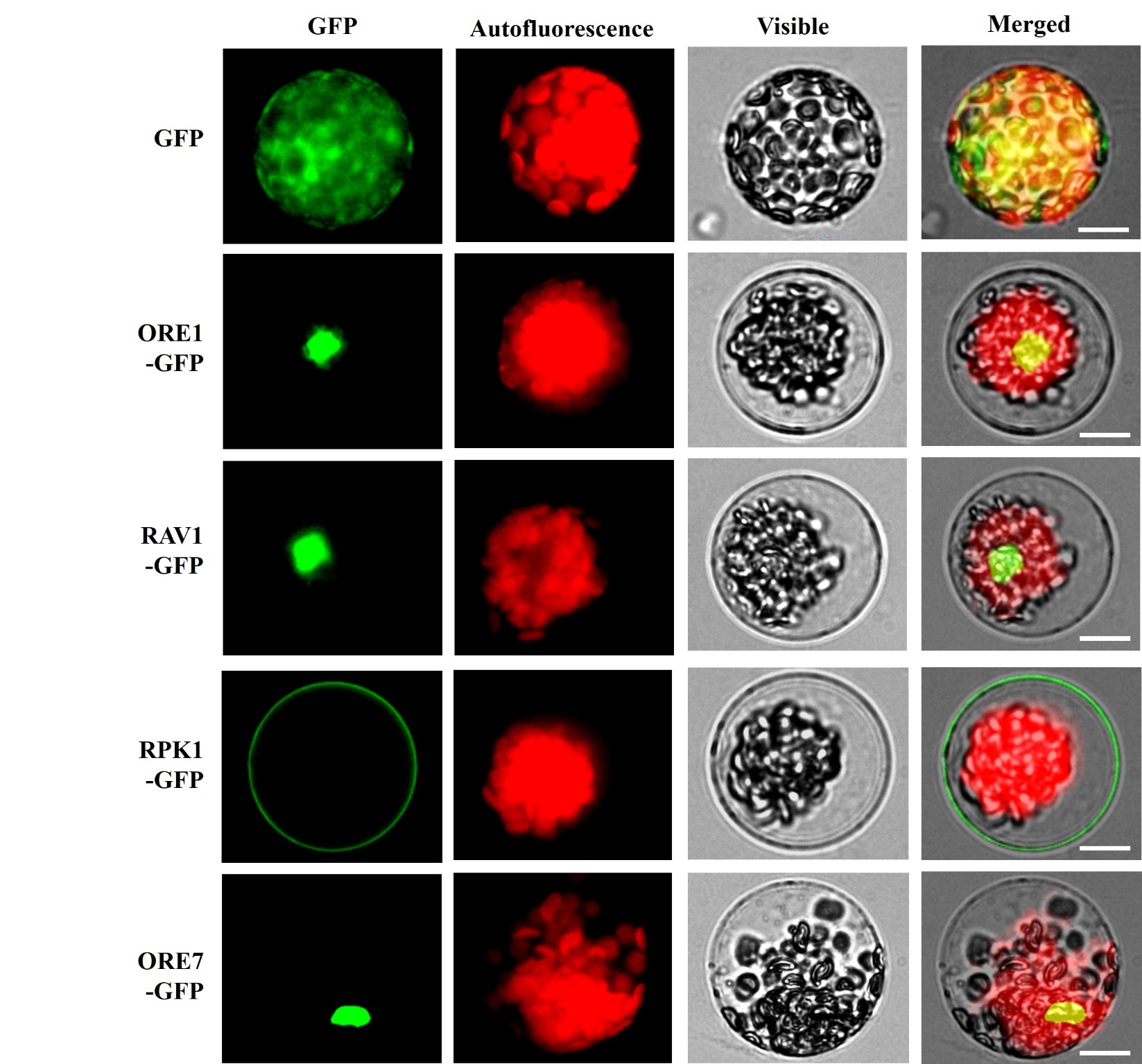


**Supplementary Figure 2.** Subcellular localization of ORE1-GFP, RAV1-GFP, RPK1-GFP, and ORE7-GFP in transiently transfected Arabidopsis protoplasts. GFP, ORE1-GFP, RAV1-GFP, RPK1-GFP, and ORE7-GFP were transiently expressed in Arabidopsis protoplast for 24h under dim white light. GFP and Chloroplast autofluorescence (red) signals were monitored by fluorescent microscopy. GFP, autofluorescence, bright-field, and the merged images are shown. Bars = 100µm.

**
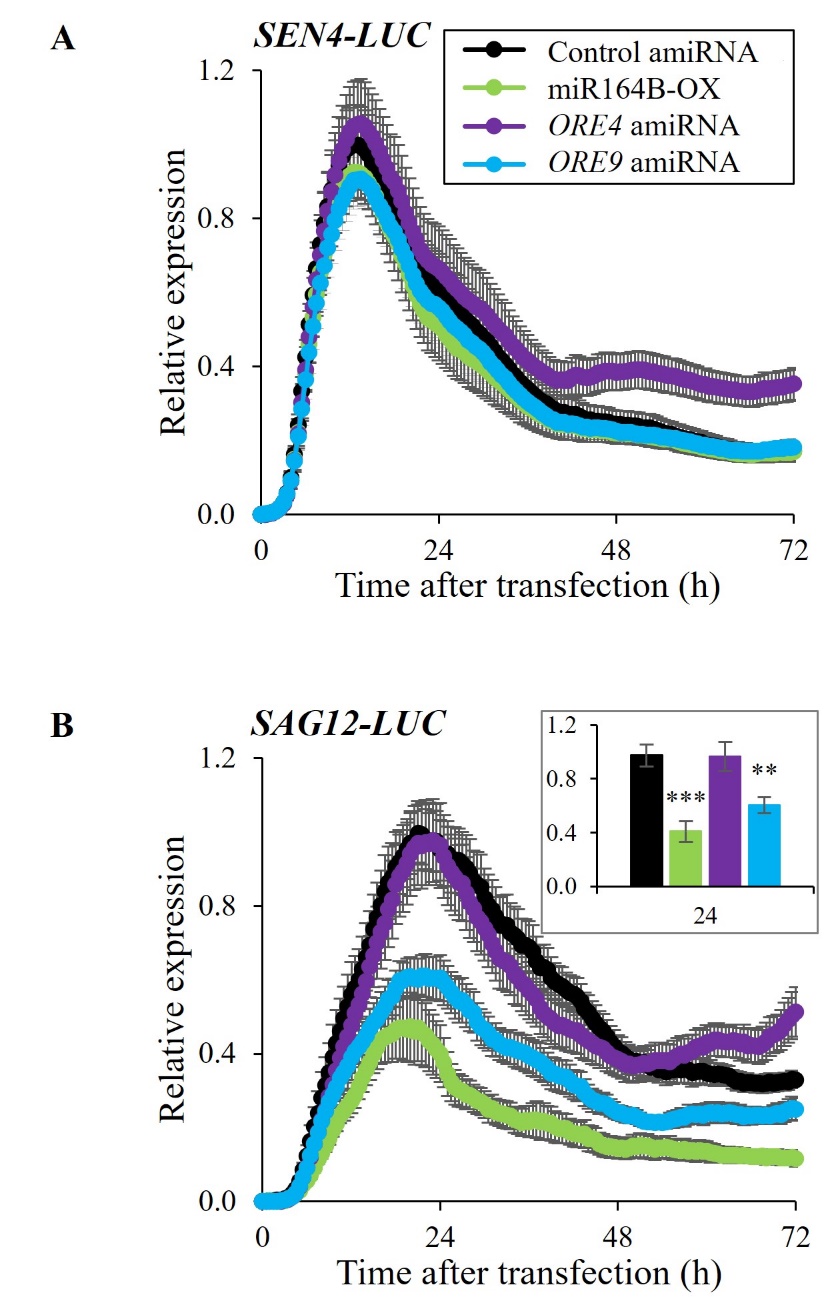
**

**Supplementary Figure 3.** Bioluminescence traces of *SEN4-* **(A)** and *SAG12-LUC* **(B)** in other amiRNAs-transfected protoplast cells. Transfection, imaging acquisition, and data processing were performed, as in **Figure 3**, but with a plasmid of miR164B-OX, *ORE4* amiRNA, and *ORE9* amiRNA as effectors. Symbols in **(B)** are the same as in **(A)**. (Inset) Bar graph of relative expression of *SAG12-LUC* at 24h after transfection. Data represent mean ± SE (*n* = 6). A statistical analysis was performed using a two-tailed Student’s *t*-test (***p,*<0.01; ****p*<0.001).


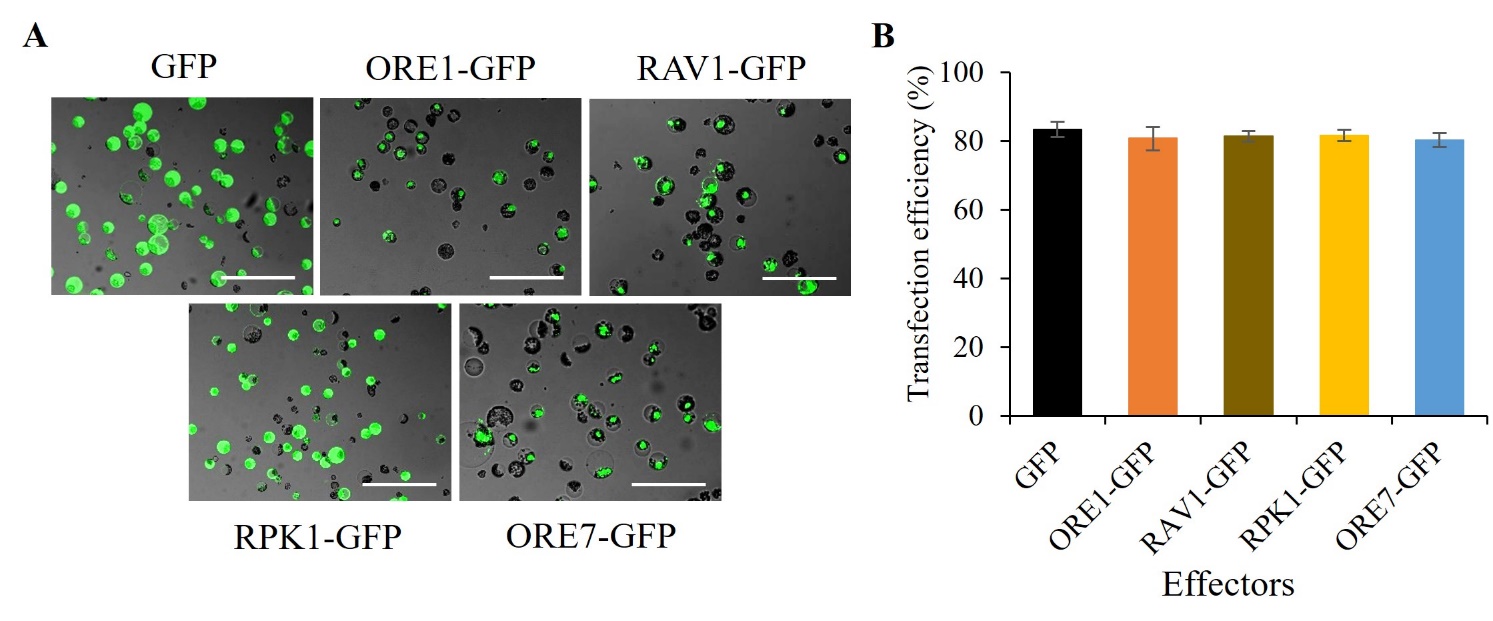


**Supplementary Figure 4.** Transfection efficiencies in Arabidopsis protoplasts. **(A)** Merged fluorescence and bright-field images of protoplasts expressing GFP-fused effectors. **(B)** Transfection efficiencies of protoplasts expressing various senescence regulators. Transfection was conducted as in **Figure 4**. GFP expression was monitored under a fluorescence microscope at 18h post-transfection. Bars = 1mm. Data represent mean ± SE (*n* = 2).


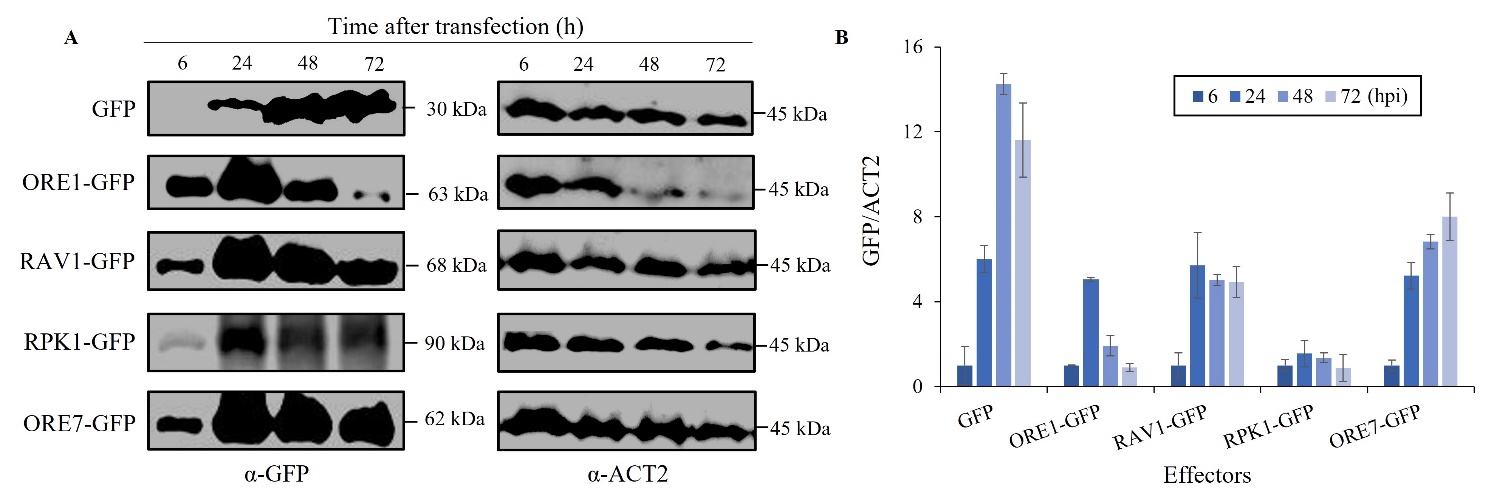


**Supplementary Figure 5.** Protein abundance of effectors in protoplasts during the assay.  **(A)** Immunoblotting of GFP and GFP-fused effector proteins in protoplasts. The figure shows expression levels of effector proteins, including GFP, ORE1-GFP, RAV1-GFP, ORE7-GFP, and RPK1-GFP in protoplasts at the indicated incubation time (h). Abundances of GFP and GFP-fused effector proteins were determined from total protein extracts of protoplast through immunoblotting. ACT2 was used as the normalization control. Data are representative of two trials. **(B)** Quantification of the relative expression of GFP and GFP-fused effectors from **(A)**. Data represent mean ± SE (*n* = 2). hpi, hours post-infection.


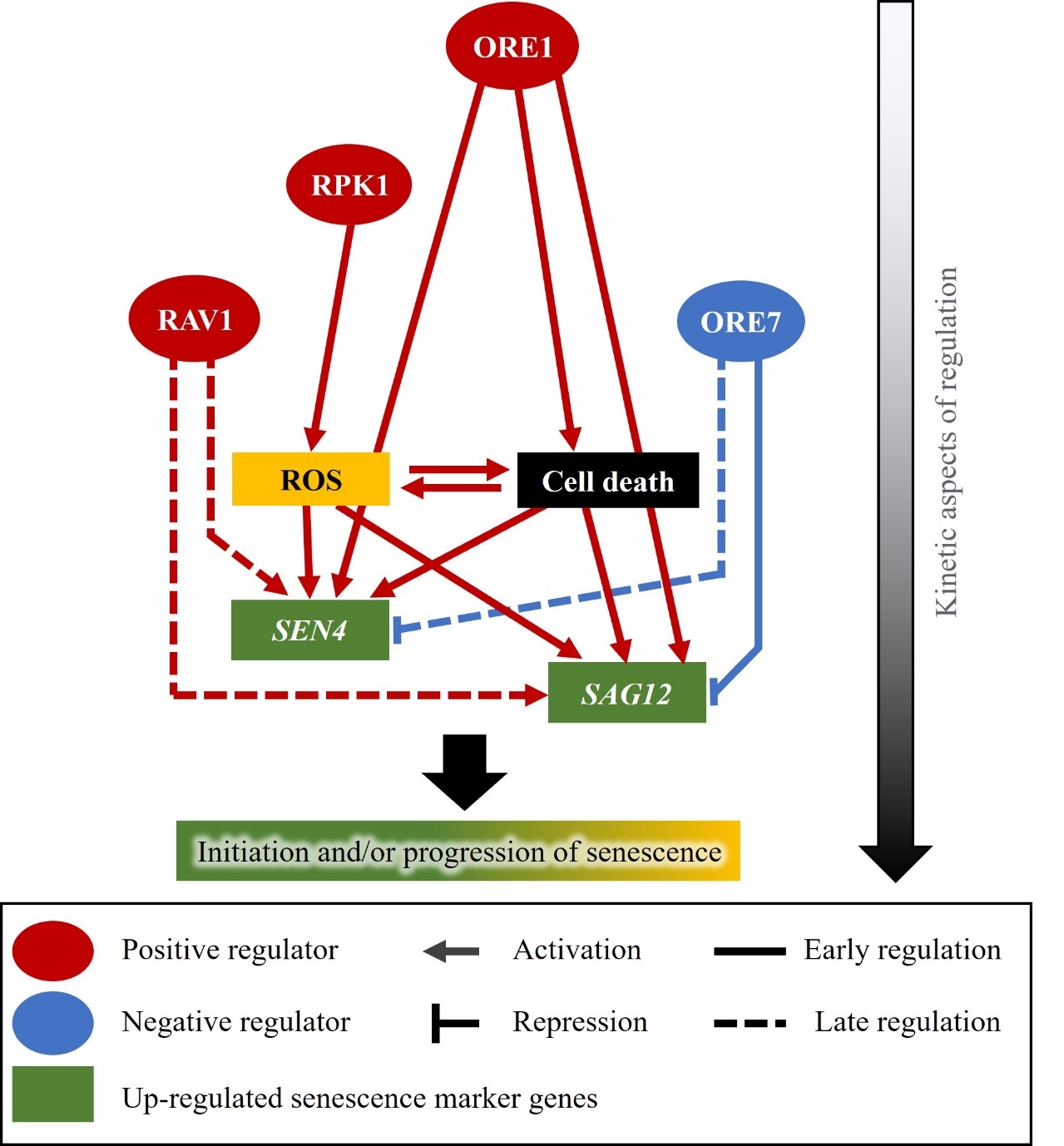


**Supplementary Figure 6.** A regulatory scheme of the cellular senescence program. In this scheme, ORE1 and RPK1 trigger senescence regulatory programs in an early senescence stage through a different mechanism; ORE1 and RPK1 mediate cell death- and ROS-induced senescence, respectively. However, RAV1 is involved in late senescence responses separately from ORE1 and RPK1. ORE7 functions as a negative senescence regulator to repress the activity of *SAG12* promoter both in early and late stage, but that of *SEN4* promoter only in the late stage.

## Supplementary Tables

**Supplementary Table 1.** **The lists of primers used in this study**

1. **Plasmid construction**
2. **Overexpression**

| Primer Name | Sequences (5’ to 3’)^1)^ | R.E.^2)^ | Vector |
| --- | --- | --- | --- |
| ORE1-F | CAAACTAGTATGGATTACGAGGCATCAAGAATC | *Spe*I | pCR-CCD-F |
| ORE1-R | CAAAGGCCTGAAATTCCAAACGCAATCCAATTC | *Stu*I |  |
| RAV1-F | TTTGGATCCGGATGGAATCGAGTAGCGTTGAT | *Bam*HI | pCR-CCD-F |
| RAV1-R | TTTAGGCCTCGAGGCGTGAAAGATGCGTTGCT | *Stu*I |  |
| RPK1-F | ATTCTGCAGATGAAACTTCTGGGTTTGGTCTTCTT | *Pst*I | pCR-CCD-F |
| RPK1-R | ATTAAGCCTACAATCTAGAAGGCTGGATTCGTTT | *Hind*III |  |
| ORE7-F | GGACTAGTATGGAAGGCGGTTACGAGCA | *Spe*I | pCR-CCD-F |
| ORE7-R | GAAGGCCTAAAAGGTGGTCTTGAAGGTGT | *Stu*I |  |

1. **AmiRNA**

| Primer Name | Sequences (5’ to 3’)^1’)^ | R.E.^2)^ | Vector |
| --- | --- | --- | --- |
| CsVMV AmiR-F | GGTGTAAGCTATTTTCTTTGAAGTAC |  | pCsVMV-PP2C-AmiR |
| AmiR nos-R | GCAACAGGATTCAATCTTAAGA |  |  |
| miR164B-F | TCTCTGCAGTTTTTGGGTAGCATGTTCAT | *Pst*I | pCsVMV-N-999 |
| miR164B-R | TCTAGGCCTCGCTAACCGAAACTATGTTC | *Stu*I |  |
| RPK1 AmiR-I | TATAAGTCGAACCGAAACCAC |  | pCsVMV-PP2C-AmiR^a)^ |
| RPK1 AmiR-II | GTGGTTTCGGTTCGACTTATA |  | pCsVMV-PP2C-AmiR^b)^ |
| RPK1 AmiR-III | GTAGTTTCGGTTCCACTTATT |  | pCsVMV-PP2C-AmiR^c)^ |
| RPK1 AmiR-IV | AATAAGTGGAACCGAAACTAC |  | a) + b) + c) |

**C. Promoter**

| Primer Name | Sequences (5’ to 3’)^1’)^ | R.E.^2)^ | Vector |
| --- | --- | --- | --- |
| SAG12pro-F | TTTGAGCTCGTTGGTACTTTGGTAGCAAGTCG | *Sac*I | pCR-CCD-R |
| SAG12pro-R | TTTGGATCCTGTTTTAGGAAAGTTAAATGACTTTTG | *Bam*HI |  |
| SEN4pro-F | TTTGAGCTCATTTGGGTTCGTATTCTTTCTCATAC | *Sac*I | pCR-CCD-R |
| SEN4pro-R | TTTGGATCCTGTCTTTGTGTGTGTGCGTACG | *Bam*HI |  |
| THIONINpro-F | TTTGGATCCATTGAAAAACAGAGGGAGTATTGA | *Bam*HI | pCR-CCD-F |
| THIONINpro-R | TTTAGGCCTCTTTTGATTGATTAGTTGTTTGATCAC | *Stu*I |  |
| CA1pro-F | TTTGGATCCCCATTCCTCGAGGTACTTAAC | *Bam*HI | pCR-CCD-F |
| CA1pro-R | TTTAGGCCTTTGTTGGCGAAGAGAAGCGGAGA | *Stu*I |  |
| PRKpro-F | TTTGGATCCCCCTTACGATATAAGGTCTGA | *Bam*HI | pCR-CCD-F |
| PRKpro-R | TTTAGGCCTTGTTGTTTGTTTGGTGTTTTGGTC | *Stu*I |  |

1) Annealing temperature for all primers is 56℃ or 55℃.

2) R.E., restriction enzyme sites.

1. **Real time PCR**

| Primer name | Sequences (5’ to 3’) | Annealing temperature |
| --- | --- | --- |
| ORE1-qPCR-F | CGTTTAGAAGGCAAATATTGTATTGA | 60℃ |
| ORE1-qPCR-R | AACACGACATATAACCCATTCGT |  |
| RPK1-qPCR-F | TGTCTCCAACGAATGTGTTTGC | 60℃ |
| RPK1-qPCR-R | GCTCGCATGGTAACCGATTAAC |  |
| ACT2-qPCR-F | CAGTGTCTGGATCGGAGGAT | 60℃ |
| ACT2-qPCR-R | TGAACAATCGATGGACCTGA |  |

**Supplementary Table 2.** **Estimates of peak time and maximum expression levels of LUC reporters in Arabidopsis protoplasts transiently transfected with a set of effector and reporter.**

**Reporter**

| Effector | Reporter  (Luciferase) | Peak time after transfection (h)^(a)^ | Normalized expression level at the peak time^(a, b)^ | Figure |
| --- | --- | --- | --- | --- |
| *GFP* | *SEN4-LUC* | 9.3 ± 0.25 | 1233 ± 515 | Figure S1 |
| *GFP* | *SAG12-LUC* | 18.3 ± 0.25 | 137 ± 35 | Figure S1 |
| *GFP* | *CA1-LUC* | 28.0 ± 1.00 | 31 ± 9 | Figure S1 |
| *GFP* | *THIONIN-LUC* | 33.5 ± 0.00 | 26 ± 2 | Figure S1 |
| *GFP* | *PRK-LUC* | 14.2 ± 2.83 | 5 ± 1 | Figure S1 |
| *GFP* | *35S-LUC* | 10.5 ± 0.50 | 1571 ± 242 | Figure S1 |
| *GFP* | Empty vector | 16 ± 1.16 | 7 ± 4 | Figure S1 |

**Overexpression effector**

| Effector  (Overexpression) | Reporter  (Luciferase) | Peak time after transfection (h)^(c)^ | Relative expression  level at the peak time^(c, d)^ | Figure |
| --- | --- | --- | --- | --- |
| *GFP* | *SEN4-LUC* | 8.83 ± 0.17 | 0.91 ± 0.02 | Figure 2A |
| *ORE1-GFP* | *SEN4-LUC* | 4.50 ± 0.00 | 1.92 ± 0.75 | Figure 2A |
| *RAV1-GFP* | *SEN4-LUC* | 25.67 ± 1.01 | 2.15 ± 0.38 | Figure 2A |
| *RPK1-GFP* | *SEN4-LUC* | 8.17 ± 0.44 | 9.61 ± 3.6 | Figure 2A |
| *ORE7-GFP* | *SEN4-LUC* | 8.50 ± 0.00 | 1.17 ± 0.16 | Figure 2A |
| *GFP* | *SAG12-LUC* | 13.67 ± 1.33 | 0.74 ± 0.09 | Figure 2B |
| *ORE1-GFP* | *SAG12-LUC* | 5.50 ± 0.29 | 0.50 ± 0.17 | Figure 2B |
| *RAV1-GFP* | *SAG12-LUC* | 35.67 ± 0.60 | 2.47 ± 0.53 | Figure 2B |
| *RPK1-GFP* | *SAG12-LUC* | 29.17 ± 1.17 | 17.53 ± 6.42 | Figure 2B |
| *ORE7-GFP* | *SAG12-LUC* | 10.17 ± 4.92 | 0.20 ± 0.08 | Figure 2B |

**amiRNA effector**

| Effector  (amiRNA) | Reporter  (Luciferase) | Peak time after transfection (h)^(c)^ | Normalized expression level at the peak time^(c, d)^ | Figure |
| --- | --- | --- | --- | --- |
| Control amiRNA | *SEN4-LUC* | 9.17 ± 0.40 | 1.00 ± 0.10 | Figure 3C |
| *ORE1* amiRNA | *SEN4-LUC* | 9.67 ± 0.28 | 1.22 ± 0.11 | Figure 3C |
| *RPK1* amiRNA | *SEN4-LUC* | 9.42 ± 0.27 | 1.04 ± 0.05 | Figure 3C |
| Control amiRNA | *SEN4-LUC* | 12.83 ± 0.21 | 1.00 ± 0.11 | Figure S3A |
| miR164B-OX | *SEN4-LUC* | 12.75 ± 0.11 | 0.92 ± 0.07 | Figure S3A |
| *ORE4* amiRNA | *SEN4-LUC* | 13.08 ± 0.24 | 1.06 ± 0.12 | Figure S3A |
| *ORE9* amiRNA | *SEN4-LUC* | 13.25 ± 0.25 | 0.91 ± 0.07 | Figure S3A |
| Control amiRNA | *SAG12-LUC* | 26.33 ± 2.99 | 1.00 ± 0.11 | Figure 3D |
| *ORE1* amiRNA | *SAG12-LUC* | 28.58 ± 8.27 | 0.34 ± 0.05 | Figure 3D |
| *RPK1* amiRNA | *SAG12-LUC* | 35.17 ± 9.71 | 0.57 ± 0.08 | Figure 3D |
| Control amiRNA | *SAG12-LUC* | 21.50 ± 0.89 | 1.00 ± 0.08 | Figure S3B |
| miR164B-OX | *SAG12-LUC* | 19.00 ± 1.08 | 0.47 ± 0.08 | Figure S3B |
| *ORE4* amiRNA | *SAG12-LUC* | 22.25 ± 0.67 | 0.98 ± 0.11 | Figure S3B |
| *ORE9* amiRNA | *SAG12-LUC* | 19.83 ± 0.99 | 0.61 ± 0.05 | Figure S3B |

^(a)^ Data represent mean ± SE (n = 2 or 3).

^(b)^ Normalized by the maximum level of RLUC throughout the measurement.

^(c)^ Data represent mean ± SE (n = 6).

^(d)^ Normalized by the maximum value of LUC/RLUC of control GFP or amiRNA.

**Supplementary Table 3.** **Calculated interaction likelihoods between amiRNAs and potential target gene(s)**

| amiRNA | amiRNA core sequence (5’ 🡪 3’) | amiRNA source^(a)^ | Potential target gene(s) | Target recognition sequence  (5’ 🡪 3’)^(b)^ | Hybridization energy (kcal/mol)^(c)^ | Perfect-match-dG cutoff (70%)^(d)^ |
| --- | --- | --- | --- | --- | --- | --- |
| ORE1 | TTAACGAAGGTAAGCCGGTG | A | ORE1 | CAGCCGGTTTACCTTCGTTAA | -40.86 | 93.76 |
| RPK1 | TATAAGTCGAACCGAAACCAC | W3 | RPK1 | GCGGTTTCGGTTCGACTTATA | -38.13 | 97.89 |
| ORE4 | TTAGTCTTACTGACGGGTCTG | A | ORE4 | AGACCCATCAGTAAGACTAAA | -36.51 | 85.34 |
| ORE9 | TCTTAGGGGAACTGGTACCTA | A | ORE9 | TAGCTACCAGTTCCCCTAAGT | -37.23 | 81.29 |

^(a)^ A, From ABRC; W3, Designed using the WMD3 (web microRNA designer3).

^(b)^ Matched sequences between amiRNA and target sequence are underlined.

^(c)^ Hybridization of amiRNA to its mRNA target site.

^(d)^ Ratio of the hybridization energy between amiRNA and its target to that between amiRNA and its perfect reverse complement in percent.

# Supplementary References

Kim, J., and Somers, D.E. (2010). Rapid assessment of gene function in the circadian clock using artificial microRNA in Arabidopsis mesophyll protoplasts. *Plant Physiol.* 154(2)**,** 611-621. doi: 10.1104/pp.110.162271.

Schwab, R., Ossowski, S., Riester, M., Warthmann, N., and Weigel, D. (2006). Highly specific gene silencing by artificial microRNAs in Arabidopsis. *Plant Cell.* 18(5)**,** 1121-1133. doi: 10.1105/tpc.105.039834.

Voinnet, O., Rivas, S., Mestre, P., and Baulcombe, D. (2003). An enhanced transient expression system in plants based on suppression of gene silencing by the p19 protein of tomato bushy stunt virus. *Plant J* 33(5)**,** 949-956. doi: 10.1046/j.1365-313x.2003.01676.x.

Yu, G., Wang, X., Chen, Q., Cui, N., Yu, Y., and Fan, H. (2019). Cucumber Mildew Resistance Locus O Interacts with Calmodulin and Regulates Plant Cell Death Associated with Plant Immunity. *Int J Mol Sci* 20(12). doi: 10.3390/ijms20122995.
